# Comparative Perspectives

**DOI:** 10.1007/978-3-030-63266-3_6

**Published:** 2021-01-21

**Authors:** Wolf Linder, Sean Mueller

**Affiliations:** 3 Department of Political Science, University of Bern, Bern, Switzerland; 4 Institute of Political Science, University of Lausanne, Lausanne, Switzerland

## Abstract

This chapter develops three comparative perspectives. Beginning with direct democracy, enhancing the direct participation of people as in Switzerland—considered revolutionary in the nineteenth century—may still be regarded a progressive form of democracy. But are increased political rights, offering the people not only a voice in electing their representatives but also a chance to decide major decisions directly, really an efficient way to improve democracy? The second perspective deals with federalism. Traditionally understood as a means for the vertical division of power within states, can it also play a role for the supranational division of power and the participation of minorities? The last section places political power-sharing in a context of conflict resolution, especially in multicultural societies. The chapter ends by stressing that power-sharing is not just an institutional arrangement, but that it also has to be based on the specific culture of a society that intends to practice it.

## Direct Democracy

### Experiences of Direct Democracy Compared

The first worldwide comparative study on direct democracy in 1978 produced some astonishing results: its authors, Butler and Ranney (1978, 7), counted more than 500 nationwide referenda in countries all over the world. Their distribution, though, was uneven. They counted 300 referenda for Switzerland, 39 for Australia, 20 for France and 13 for Denmark. In all other countries the number was below ten. Forty years later, Qvortrup (2018, 264) counted already 331 nationwide referendums in democratic polities between 1900 and 2017 *without* Switzerland’s 556 votes in the same period. Table 6.1 provides an overview over the past 70 years.

**Table 6.1:** Number of nationwide referenda, 1950–2019

Area	Number	Share
Europe (excl. Switzerland)	552	30%
Switzerland	479	26%
Australasia and Asia	265	15%
Africa and Middle East	259	14%
Americas	256	14%
*Total*	*1811*	*100%*

Including post-colonial independence votes if successful. Data from www.sudd.ch, 19.12.2019

Concerning the issues of votes, one can distinguish three general categories. The first one comprises the establishment or secession of a state, of a new constitutional order or regime. In these cases, the principle of self-determination of a people, and the attempt to provide legitimation for fundamental changes in the political order are important motives. Some historical examples are the separation of Norway from Sweden in 1905, the vote of English Togo (under UN supervision) to join Ghana and of French Togo to become independent in 1965, or the case of the Philippines where, in 1986 after the end of the Marcos regime, President Corazon Aquino allowed the people to ratify the new Constitution. At the beginning of the 1990s, the transformation of communist regimes to democracies in Eastern Europe saw many referenda in Kyrgyzstan, Azerbaijan, Turkmenistan and in the Baltic states. In the reunion of Eastern and Western Germany, however, the peoples were not granted a say.

A second category, relatively new, comprises decisions on membership in transnational organisations or changes in the status of such membership. In both cases, votes are held because the member states agree to share part of their sovereignty with the trans or supranational organisation. Spanish citizens, for instance, voted to remain in the North Atlantic Treaty Organization (NATO) in 1986. On EU issues, Denmark has so far held eight and Ireland nine referenda. In 2005, the people of France and the Netherlands famously voted against the adoption of the European Constitution. Before the EU enlargement of 2004, referenda were held in nine out of ten candidate countries (Slovenia, Czech Republic, Slovakia, Estonia, Latvia, Lithuania, Hungary, Poland and Malta; Szczerbiak and Taggard 2004; C2D 2019). The most recent example, of course, is Brexit: in June 2016, a majority of UK voters decided to leave the EU.

A third category deals with important national policy decisions for which a government wants to be given additional legitimacy. Chapter 10.1007/978-3-030-63266-3_3 already mentioned French President de Gaulle’s plebiscite on Algerian independence in 1961, which put an end not only to the colonial regime but also to the deep divide of the French nation on this question. In some East-European countries, plebiscites were used from the very beginning of the liberalisation process. Whereas the Polish authorities failed to obtain the support of the people when trying to pass early reforms for economic liberalisation, the Hungarian opposition in 1989 won a referendum on the question of election procedure against the wishes of the still communist-controlled government.

These examples illustrate the vast variety of occasions on which people are able to express their preferences. For a better understanding of the different uses made of the devices of direct democracy, a classification according to the following criteria is useful:

A. *Binding versus non-binding* *referenda*: It is obvious that binding referenda have a higher impact than non-binding ones which are merely consultative or advisory (cf. also Cheneval and el-Wakil 2018, 300). In New Zealand, for instance, the referendum is non-binding and it is left to the government or legislature to interpret the results. For binding referenda, the consequences depend much on the type of the popular vote.
B. *The authority empowered to call a popular vote*: With regards to who has the authority to demand that a popular vote be held, we can distinguish four basic types:
  1. Government-controlled: The majority of parliament or the president have the sole power to decide whether or not a referendum is held. They decide the subject matter and the wording of the proposition to be voted upon. This type is often referred to as a plebiscite.
  2. Constitutionally required: The Constitution requires that certain decisions ( constitutional amendments, ordinary laws, decisions on financial or international issues) be approved by the voters before they take effect. The government might still have a free hand in formulating the proposition, but is legally bound to a direct-democratic procedure.
  3. Referenda called by the people: A certain number of voters are authorised to demand a popular vote be held on specific government decisions, either before or after these have taken effect. Thus, it depends on a group of citizens to decide whether a government decision has to be ratified by the people. A similar device is the recall, which allows a certain number of voters to demand the removal of an authority or a single person from office.
  4. Popular initiatives: A certain number of voters are authorised to demand a popular vote on broad statements of intent or specific measures which they themselves have proposed. Thus, it is a group of people who, acting as ‘lawmakers’, decide the subject matter and the wording of the proposition to be voted on.^1^

Most countries know only the first type, the plebiscite. Under such an institutional arrangement, direct democracy is limited in use and purpose. If it is left to the discretion of the government to put issues before its voters, the referendum tends to serve as an occasional device to obtain wider support for a presidential or parliamentary policy. This is especially the case with non-binding plebiscites, in which the government can realise its projects also if defeated in the vote. A special case is the UK’s vote on its continued EU membership held in 2016 ( Brexit), which although merely advisory the government had promised to honour whatever the outcome.

Types (2)–(4) are fundamentally different from plebiscites. In those cases, a pre-defined class of government decision is always subject to a constitutionally required (mandatory) referendum; and citizens can, by petition, challenge government decisions (optional referenda) or even hand in their own proposals for constitutional or legislative reform ( popular initiatives). The difference is that all these devices sanction or correct government policies and politics even when the government might *not* wish for popular interference. Under these institutional arrangements, direct democracy thus gives citizens an independent voice in politics and policies. This may be in accord with governmental policies, especially in the case of constitutionally required referenda. But the voice of the citizens can be, and often is, also raised against the government. To challenge government decisions in a selective way is the ‘natural’ use of popular referenda. The idea of ‘correcting’ representative democracy is further developed by the popular initiative, which allows the people to not only approve or reject government decisions, but also offers a group of citizens the chance to have their own propositions put to a popular vote.

The list of countries where direct democracy is used to challenge or correct the parliamentary process is short. In Australia national referenda, which are required for certain constitutional amendments, are held quite frequently. The Italian Constitution provides for referenda with the proviso that citizens can challenge a parliamentary law only sometime after its introduction and application. This unique ‘abrogative referendum’ was used in the divorce issue for instance, when part of the Catholic population wanted to abolish the secular and liberal divorce law. The Philippine Constitution of 1986 has institutionalised both the initiative and the referendum. Recently, Slovakia, Hungary, Lithuania and some South-Caucasian states have introduced referenda on constitutional reforms.

A final distinction separates *national from sub-national* *referenda*. While in Switzerland direct democracy is known on all three federal levels, other countries practice direct participation only on the sub-national levels. This is the case, for instance, in Germany were votes are held in some *Bundesländer* and their communes. A prominent case are the US States, where direct democracy is as widely institutionalised and used as it is in Switzerland. In all US States with the exception of Delaware, any amendment to the State constitution requires a popular vote. In about half the States we find one or another type of referendum for parliamentary laws, often complemented by a financial referendum. Moreover, citizens in many States can propose legislation by means of the popular initiative, or initiate a ‘recall’, which allows voters to remove or discharge a public official from office. In no other part of the world but California have citizens had so much opportunity to express their political preferences: from 1884 to 2018, Californians voted on more than 2000 issues.^2^

### The Practice of Direct Democracy in US States and Switzerland: Similarities and Differences

US direct democracy is fundamentally different from Switzerland’s in one point: it is limited to the sub-national level. Populist forces in the late 1970s demanded nationwide referenda without success. They had no real chance to change the tradition of republican belief in the system of ‘checks and balances’, which is opposed to any form of plebiscite at national level. Yet, the US States’ and Switzerland’s experience of direct democracy are the richest: the instruments of the referendum and the popular initiative are practically the same, and one can find many similarities in their use. For an assessment of direct democracy, it may thus be most useful to compare their experiences.

In his overall appraisal of direct democracy in US States, Cronin (1989, 222) comes to the following conclusion:

In sum, direct democracy devices have not been a cure-all for most political, social, or economic ills, yet they have been an occasional remedy, and generally a moderate remedy, for legislative lethargy and the misuse of legislative power. It was long feared that these devices would dull legislators’ sense of responsibility without in fact quickening the people to the exercise of any real control in public affairs. Little evidence exists for those fears today. When popular demands for reasonable change are repeatedly ignored by elected officials and when legislators or other officials ignore valid interests and criticism, the initiative, referendum and recall can be a means by which the people may protect themselves in the grand tradition of self-government.

This assessment could also be largely subscribed to in the case of Switzerland, whose ideas of popular control of representative government in fact influenced the development of direct democracy in the US between 1890 and 1920 (Auer 1989). Another common conclusion can be drawn: historically speaking, critics as well as proponents of direct democracy overestimated the power of the referendum and the initiative, whether for ill or good. Finally, even if voters in the US and Switzerland are aware of its limited effects and deficiencies, direct democracy constitutes an element of political culture that citizens are unwilling to relinquish.

Further similarities show up when comparing a number of Cronin’s (1989, 224–32) points on the ‘general effects of direct democracy devices’:

1. *Uncertainty on the question if ‘direct democracy can enhance government responsiveness and* *accountability’*. For Switzerland, we have noted several characteristics of the public sector (the small budget of central government, limited public administration, the modification of a proposed policy programme after its defeat in the first popular vote, etc.) that indicate a high level of responsiveness to the ‘will of the people’. On the other hand, the power-sharing coalition of an all-party government can also work as a political cartel and thus reduce responsiveness. Valid comparisons, though, cannot be made. In the US, where comparison with purely representative States is possible, Cronin notes that ‘few initiative, referendum and recall States are known for corruption and discrimination. Still, it is difficult to single them out and argue persuasively that they are decidedly more responsive than those without the initiative, referendum, and recall’.
2. *As in Switzerland, ‘direct democratic processes have not brought about rule by the common people’*. In both systems, more than 90% of important parliamentary decisions are not challenged. Popular initiatives alter and influence the political agenda, but do not call into question the role of parliament as the chief lawmaker. At more than 45%, the rate of successful initiatives is higher in the American States than in the Swiss federation (10%) and its cantons (30%) (Linder and Mueller 2017, 328). But in both countries direct democracy is fraught with inequalities in participation. It is the better educated, older and financially better-off citizen who engages and participates significantly more in direct democracy. Empirical data indicate that the more complicated the procedure and the issues at stake, the more direct participation is socially discriminatory. This selective bias affects the devices of direct democracy, whose specific policy ramifications can be much harder to grasp than simply casting a vote for a person or party based on sympathy or habit (see Chap. 10.1007/978-3-030-63266-3_4 and Cronin 1989, 76). Finally, direct democracy requires citizens to get organised. Cronin states that ‘direct democracy devices occasionally permit those who are motivated and interested in public policy issues to have a direct personal input by recording their vote, but this is a long way from claiming that direct democracy gives a significant voice to ordinary citizens on a regular basis’.
3. *‘Direct legislation does not produce unsound legislation and unwise or bad policy’*. There are strong arguments for this value judgement, despite empirical evidence in both countries that citizens are not always well informed about the issues on which they vote. For the Swiss case, Kriesi’s (2005) analyses show that simplifying strategies such as heuristic voting based on cues or party recommendations do not lead to irrational choices. For the US case, Cronin states that the contributions of direct democracy do not essentially differ from those of parliament. As with every procedure based on majority rule, minorities can lose, and this risk, according to Cronin, may even be slightly greater under direct than representative democracy. The same can be said for Switzerland, where recently three popular initiatives gave rise to questions about their compatibility with the Constitution and fundamental rights (cf. also Christmann and Danaci 2012). But voters in direct democracies ‘have also shown that most of the time they too will reject measures that would diminish rights, liberties, and freedoms for the less well-represented or less-organized segments of society’ (Cronin 1989, 123).
  Kriesi’s and Cronin’s arguments, however, compare only direct and parliamentary legislation. How about the fundamental question: does direct participation lead to more or less democratic stability? The quality of direct democracy will depend on the consolidation and quality of democracy as a whole. Even for the consolidated case of Switzerland, there is empirical evidence that direct democracy is ambiguous. On the one hand, it has integrating effects. On the other, it allows political elites to use fundamental societal cleavages for mobilising voters. The latter effect may be detrimental for an unstable, not-yet-consolidated democracy. Germany’s regression from a democracy to an authoritarian regime was ‘legitimated’ by three plebiscites in 1933–1938, and Austria too approved its *Anschluss* in a popular vote. If Switzerland at that time rejected the popular initiatives of the Frontist movement, an important reason for this was that besides the people, a clear majority of the political elite was also hostile to the idea of fascism (Neidhart 1970, 238–43). These historical examples illustrate that direct democracy is vulnerable: instead of contributing to political integration, it may be a factor of de-stabilisation in deeply divided societies and unconsolidated democracies (Linder et al. 2008).
4. *‘Direct democracy can influence the* *political agenda* *in favour of issues important to less well-organized interests’*. Environmentalists provide a good example of this for California and Switzerland. The popular initiative widens the political agenda and the horizon in respect of what is politically conceivable. We have to note, however, that these innovative effects may become unwelcome. In California, for instance, there is criticism that direct democracy is part of the reason why the state has become ‘ungovernable’: an abundant number of popular initiatives is launched by a professional campaigning industry that promotes special vested interests rather than those of the ordinary citizens (The Economist 2009). In Switzerland, the smaller ‘political market’ and lower success rates of popular initiative may have set closer limits to a professional referenda industry.
5. *‘Direct democracy tends to strengthen single issue and* *interest groups* *rather than political parties with larger, general interest, programmes*’. Popular democratic rule partially loses or changes its meaning when devices of direct democracy, originally used by social movements, pass into the hands of interest groups (Hofstadter 1955, Croly 1914; also Bühlmann and Kriesi 2007, Schneider and Hess 1995, and Schneider and Weitsman 1996). The ‘normal’ form and function of direct democracy are not what they were at the beginning. This statement for Switzerland can be complemented by the US experience that ‘Initial achievements or victories were won by the populists and progressives, but the very bosses or interests against whom these devices were aimed soon learn to adapt to the new rules, deflect them, or use them to advance their strategic interests’ (cit. in Cronin 1989, 231). Yet Cronin, who partially agrees with this critique made by both Croly and Hofstadter, also emphases that special interest and single-issue groups regularly take part in both direct as well as representative democracy . If the US has become a nation of interest groups , it is the very task of politics to blend divergent interests into great governing coalitions. This, in Cronin’s view, parliament is best placed to achieve.
6. *‘Money is, other things being equal, the single most important factor determining direct legislation outcomes’*. It costs money to collect signatures for a referendum or initiative, to create and maintain an effective campaign organisation, to formulate and pass a political message on to voters by direct mail, to finance propaganda and attract the attention of the mass media. The frequent use of the devices of direct democracy has led to the professionalisation of campaigns, an evolution well known in the US and observable also in Switzerland, albeit with a time lag. Unequal distribution of money leads to unequal campaign spending, sometimes up to ratios of 1:20 or 1:50. In Switzerland as in the US States, the high-spending side wins in many cases, yet only in the US do strict rules on financial transparency exist (Garret & Smith 2005). It is exceptional for underdogs to win against ‘big money’. Some American scholars speak of campaign money as the single most powerful predictor of who wins and who loses (Zisk 1987, 90–137; Loewenstein 1982, 505–641). In the Swiss case, there is evidence that money cannot play the same role with all votations (Kriesi 2009). In the case of pre-dispositioned issues, where citizens’ preferences are related to first-hand experience and their own values, campaigns have less effect than on non-pre-dispositioned, mostly complex and abstract issues. Moreover, money is absorbed into political parties’ campaign strategies, which include not only propaganda but also ‘argument based’ reasoning to convince voters. Votations cannot generally be bought. But on highly controversial questions with heavy campaigning because of an expected tight vote, money can be the decisive factor (see Sect. 10.1007/978-3-030-63266-3_4#Sec21).To a certain extent, money can be substituted by voluntary work of political activists. Together with socially unequal participation, however, the distorting effect of money remains probably one of the most serious deficiencies of direct democracy. First, unbalanced campaign spending devaluates the fundamental idea of a democracy based on ‘one person, one vote’. We could draw an analogy with a town meeting or a television debate where one side gets to speak twice, five or 20 times more often than the other side. Second, the risk of deceptive advertising can be greater if there is no counterbalance. Citizens can be prevented from making a fair judgement of the real issue. These deficiencies, however, are not specific to direct democracy. The distorting influence of money (and the media more generally) can also be observed during elections in representative systems, as regular discussions in the US show. The money question is as unresolved in Switzerland as in the US, where attempts to regulate the financing of direct-democratic campaigns have been thwarted in the courts.

After all these similarities, there are three main differences:

1. *In the US States, direct democracy is not an element of political power-sharing* . With their two-party systems, winner-take-all elections and relatively homogeneous majorities installed by a white Anglo-Saxon Protestant hegemony, the referendum has not become a device to permit cultural minorities—African Americans or indigenous peoples, for instance—to gain better access to power or achieve proportional representation. Nor do we know about negotiation processes carried out in the shadow of the referendum challenge, which so much characterise Swiss decision-making. One reason for this might be that US interest groups find it much easier to exert their influence through parliamentary bargaining. Lobbyists in the US legislative tradition can try to get their interests to appear in many bills by attaching their desires as ‘riders’ (non-germane amendments). This leads to bills that are sometimes a conglomerate of matters such as money for agriculture, schools, highway construction and so on. Non-germane amendments facilitate the finding of ‘ constructive majorities’ between interest groups. In Switzerland—as in other European legislative traditions—these deals would not be possible because different matters must be regulated by different bills. In the US, however, they allow interest groups to influence legislation in a direct way without the ‘referendum threat’, which anyway is riskier. US States’ direct democracy, therefore, is neither an incentive for cooperation and power-sharing as in Switzerland, nor does it have the institutional function of political integration. In turn, because of the strong two-party system, US direct democracy has not devalued elections as the mechanism of government-selection as much as in Switzerland.
2. *Direct democracy in the US complements the representative polity, while in Switzerland it has transformed the entire political system*. With the introduction of the referendum in 1874, Swiss political institutions—which originally followed both representative *and* majoritarian ideas—were completely restructured. Majoritarian democracy was transformed into a system of consensus democracy. Negotiated legislation, compromises and permanent power-sharing became necessary if the government was to avoid defeat in referenda. This institutional transformation has not happened in the US. Especially the idea of proportional representation seems to contradict American political culture , which favours competitive elections and ‘clear’, that is, majority decision.^3^ To the Swiss observer it seems as if representative and direct democracy in the American States were much more independent of each other. In terms of political culture, the predominant ideas in Switzerland are participation and voice, while in the US they are competition and victory.
3. *In one respect direct democracy is of much greater consequence in Switzerland than in the US*. The referendum and the popular initiative are also used at national level. This distinction is important. In Switzerland, not only national but also foreign policy issues can become the object of direct democracy. The latter is even more astonishing as the Swiss Constitution was influenced by nineteenth century doctrines which put foreign policy firmly into the hands of the executive so that it has complete autonomy in its dealings with other nations. In practice, the Federal Council is under much less parliamentary control for its foreign policy than for domestic affairs (Kälin 1986). Three constitutional amendments, passed in 1920, 1977 and 2003, introduced and further extended the people’s rights in foreign policy. Today, membership in international organisations and all international treaties implying substantial unifications of law are subject to mandatory referenda (Aubert and Mahon 2003, 1102–20; Häfelin et al. 2016). If the government should want Switzerland to become a member of a supranational organisation such as the EU or a system of collective security such as NATO, a referendum is obligatory. The Swiss polity thus empowers the people to participate in matters which used to be the sovereign right of the monarch in earlier times and which have largely remained the prerogative of the executive in most other states (Delley 1999).

### The Theory of Direct Democracy: Between Ideal and Reality

#### Direct Versus Representative Democracy

In the US, where the development of modern democracy was accompanied by theoretical debates among the Fathers of the Constitution, the two different strands of direct and representative democracy were present right from the start. On one side were Benjamin Franklin and Thomas Jefferson, suspicious of government but confident of the common sense of the people. Jefferson, especially, held that the will of the people was the only legitimate foundation of government, and ‘wished to see the republican principle of popular control pushed to its fullest exercise’ (Cronin 1989, 13). On the other side, John Adams and James Madison, advocates of informed, wise and responsible decision-making by elected representatives, were sceptical about possible abuses of democracy by an ill-informed, irrational general public. The US Constitution, as a purely representative system with its checks and balances and filters such as the—nowadays purely formal—indirect election of the president, much resembles this model of prudence. Representative government, besides having become the standard all over the world, serves as a normative reference point in much democratic theory of today. And many of the arguments against direct democracy have not changed much since Madison’s times: participation beyond elections transcends the horizon and competence of most people, who are not willing to engage in or spend much time on the study and discussion of complex public affairs. The building of consensus, they say, should be left to political elites.

The case for direct democracy in modern theory, as represented by Benjamin Barber (1984) and others, can be made on two grounds. The first argument is a critique of the representative model: if representative government is more than an elitist power arrangement, its elected officials must somehow be *responsive* to their constituency. But on this point the theory of representative democracy was never clear. The debate between ‘mandate’ (elected representatives have to present their voters’ views as faithfully as possible) and ‘independent’ theorists (the representative’s duty is to deliberate free from particular interests and in the general interest of all) is still unresolved. The ambiguity and weakness of the representative model—‘thin democracy’—can be remedied only through the direct participation of the people to produce a ‘strong democracy’ (Barber 1984).

The second argument concerns the role of democracy in and for society. Whereas part of modern theory—especially economic theory, beginning with Joseph Schumpeter (1942) and Anthony Downs (1957)—considers democracy merely as an instrument for choosing the governing elites, populist-plebiscitary proponents share the unbroken tradition of a broader normative concept: democracy has to *liberate* women and men alike. Democracy as citizens’ deliberative involvement and participation in public affairs becomes part of an individual’s social and individual self-development and creates citizenship and political community (Barber 1984, 179ff.; Rosenberg 2007; Dryzek 2002).

#### ‘Sensible’ or ‘Semi-Direct’ Democracy: A Third Model?

The sharp contrast between models of direct and representative democracy disappears when looking at actual practice. Despite the many weaknesses in the theoretical model, representative government has become the predominant type of democracy. Competitive elections with the real possibility for a change in power seem to be responsive enough, at least in economically developed countries, to work satisfactorily for most citizens. Democratic government ‘for’ the people is realistic in the sense that a large majority of citizens are not, and probably will never want to be, political activists—or ‘vulcans’, as Brennan (2016) calls the ‘ideal-type’ voter.

But in some democracies, such as in the US States and in the Swiss federation and its cantons, citizens wanted more. It was the deficiencies of representative government as well as the citizens’ claim for personal expression and political participation that gave populist movements their successes when introducing the devices of direct democracy into initially representative systems. The experiences of this amalgam have dashed the original hopes of populists and contradicted most of the fears of elitists—at least in practice. Regarding the debate between proponents of direct and indirect democracy, the predictive value of democratic theory has been rather disappointing, except for one important point: direct democracy, by giving people the power to define when and on which issue to take things into their own hands, has always acted as a corrective to representative government.

In the view of Thomas Cronin, this amalgam of representative government and corrective direct democracy constitutes a third model, sensible democracy or ‘semi-direct democracy’ in the case of Switzerland. This model is realistic in a double sense. It reminds us that on a large scale, direct democracy is only feasible in combination with representative government. And, as a supplementing element, its effects on policies and political processes should not be overestimated:

Sensible democracy, with its referenda, initiatives and the recalls:

1. Values representative institutions and wants legislators and other elected officials to make the vast majority of laws;
2. Values majority rule yet understands the need to protect minority rights most of the time;
3. Wants to improve legislative processes;
4. Wants occasionally to vote on public policy issues;
5. Wants safety-valve recall or vote-of no-confidence procedures as a last resort for inept and irresponsible public officials—but is willing to make these options difficult to use;
6. Wants to improve the ability of the ordinary person both to run for office and to use direct democracy procedures;
7. Wants to lessen the influence of secrecy, money, and single-interest groups in public decision-making processes;
8. Trusts representatives most of the time, yet distrusts the concentration of power in any one institution;
9. Trusts the general public’s decision some of the time, yet distrusts majority opinion some of the time;
10. Is indifferent to most initiatives and referenda except when it comes to its own pet initiative issue;
11. Agrees with the central arguments of both the proponents and opponents of populist democracy, hence favours a number of regulating safeguards for direct democracy devices;
12. Is fundamentally ambivalent toward popular democracy—favouring it in theory and holding a more sceptical attitude toward it as it is practiced in states and localities. (Cronin 1989, 249–51)

Taking into account the slightly different experiences of Swiss semi-direct democracy, four points deserve closer scrutiny. All are based on the central argument that relations between direct democracy and representative government can also develop in a less harmonious way than argued by Cronin:

1. *Participation and the problem of social* *equality*: As mentioned earlier, direct democracy is particularly sensible to the unequal participation of citizens, and to the inequality between different groups in gaining the attention of the public at large and in influencing public opinion. Under these conditions, point 7 of Cronin’s list may be too optimistic. As Macpherson (1977) mentions, it is hard to escape a vicious circle of the sort that better participation first needs more social equality—and that more social equality in turn requires better participation. Whenever democratic theory makes its normative point about equality in society (Dahl 1989, 323ff.), it rests mostly on a moral appeal that is unconvincing because of its essential point that democratic procedures *by themselves* have an equalising effect. In practice, sometimes they do, sometimes they don’t. Neither the model of direct nor that of sensible democracy provides a convincing answer.
2. *Normative orientation*: Cronin’s model of sensible democracy does not imply that certain subject matters be excluded from the people’s vote. In his concluding remarks, however, he opposes national referenda and initiatives being held in the US, among other reasons on the ground that ‘too many issues at the national level involve national security or international economic relations’ (Cronin 1989, 251). We encounter here one of the discrepancies (nr. 12 of his model) between theory and practice. In practice, Cronin makes a good point: military power and negotiation of global terms of trade, on which the ‘way of life’ of US people depend, may be better left in the hands of a strong presidency and Congress. Thus US citizens, renouncing on direct participation at the national level, may make a rational choice as long as they prefer benefitting from international strength and supremacy. Theoretically, however, there is no reason why the model of ‘ sensible democracy’ should not also apply at national level—at least in domestic affairs.
3. *Optimal influence of citizen preferences*: The term ‘ sensible democracy’ suggests that institutional arrangements are such that the preferences of citizens have the utmost influence on government politics and policies. Sensible democracy, complementing representative decisions with occasional popular votes, seems to fulfil this criterion. But it depends on additional specificities of the institutions whether the optimum influence of citizens can be achieved, and sensible democracy has many forms. Taking first the Swiss case, we observe a high interdependence between representative and direct-democratic procedures. Because direct democracy is also a means for the political opposition, the referendum challenge enforces legislation by negotiation and power-sharing. As discussed in Chap. 10.1007/978-3-030-63266-3_5, proportional representation can devalue elections, however. As to the responsiveness and sensibility of government, there is a clear trade-off between elections and voting: Swiss citizens lose in ‘programmatic control’ through elections what they win in ‘issue control’ through direct democracy. Thus, empirical evidence casts some doubts on whether *any* combination of direct democracy and representative government can always give citizens optimal influence. Second, there may be other models. Fritz Scharpf, in his *Democratic Theory* (1970, 54ff.), provides some strong arguments in support of the idea that enhancing participation in practice leads to a group pluralism that favours the status quo of ‘haves’ and which eliminates basic reform issues that ‘have-nots’ need most. He therefore proposes a model that maximises voters’ preferences through elections, the simplest and socially least discriminatory mechanism. According to Scharpf, the system most responsive to voters’ preferences for structural reform is given by a two-party parliamentary democracy sensitive to small electoral changes, with enough power to overrule resistance by pluralist interest groups. Consequently, Scharpf puts priority for enhancing participation not in the field of political institutions but with society and the economy.
4. *Population size—a limiting factor for* *sensible democracy?* Historical experience provides evidence that semi-direct democracy may work not only in a small, 8.6 million country like Switzerland but also in California with a population of some 40 million. But could the practice of referenda and initiatives also work nationwide for the US with over 300 million, or India with 1.3 billion inhabitants? The idea is regarded by many with scepticism, yet the reasons remain vague. Is direct democracy the most vulnerable part of democracy in large countries because of increasing manipulation by big money and the mass media? Or is direct democracy an appropriate way to make central government more responsive? Nobody knows, but one point seems clear: the political culture of direct participation is a collective learning process that needs time to develop, as well as possibilities to correct errors. From this perspective, a bottom-up development from the local via the regional to the national level seems more appropriate than top-down imposition, both in respect of democracy and direct participation .

#### Perspectives of Direct Participation

Sensible or semi-direct democracy , the amalgam of parliamentary decision-making by way of referenda and popular initiatives, is not the only way to give people a say beyond elections. In the last decades, direct participation has made its way in different forms from the local up to the national level. If in European countries nationwide plebiscites and votes on EU-affairs have become more and more frequent, this may be seen as the result of strong grass-roots movements that started half a century ago. Civil rights movements in the US , and students and many other populist movements in European countries were dissatisfied with the lack of government responsiveness, challenged elitist politics and claimed more political participation. New social movements, grassroots politics and non-governmental organisations have made civil society more active in daily politics. Instruments of direct participation, especially at the local and regional levels, have developed in many forms, including advocacy planning, citizens forums, participatory budgeting, panels or citizens networks, to mention just a few (e.g. Ekman and Amnå 2012). With the development of the internet, the range of mobilisation has drastically increased. The local and the global are more and more interconnected (Tarrow and della Porta 2005).

Direct participation also plays a role for young democracies. In Central and Eastern Europe, we find experiences with direct democracy despite a difficult situation: having to walk the arduous path of developing a civic culture, democratic institutions and a market economy all at the same time. In Brazil or South Africa, landless workers’ and farmers’ movements are claiming their rights through combinations of direct confrontation and negotiation with government. Decentralisation projects in sub-Saharan countries often go hand in hand with the direct participation of locals in planning and budgeting, including procedures allowing even illiterates to participate. In these cases, direct participation allows for more than people expressing their needs—it is also a device to make people familiar with the functioning of the local state and democracy (Linder 2010).

All these experiences of direct participation, made in completely different contexts, have some characteristics in common. They are still at an experimental stage, punctual if not exceptional, and they are able to influence institutional politics only in a modest way. Even so, they all are driven by the motives of people to have better voice for their values, interests and rights, which may lead to sustainable forms of participative democracy eventually.

It would be wrong, however, to see more participation as the only means of improving democracy, or to hope that direct democracy will provide the answer to all problems of governance. Governing also always implies making decisions for groups and interests which cannot be democratically represented, and which cannot adequately participate. Decisions about the education system, for instance, mostly affect young people who cannot vote yet but are made by adults. Many social reforms, such as of criminal law or psychiatry, need the advocacy of professionals, journalists and other members of an ‘active public’. Most importantly, all societies have to take account of future generations. Especially people living in highly industrialised democracies are consuming in a few decades natural resources that took millions of years to develop. Ever-increasing energy consumption and CO_2_-emissions have become a threat to the climate itself. Such long-term effects of industrial activity are neither integrated into the price system of the market nor taken care of in today’s democratic procedure. Can we think of finding democratic majorities for decisions renouncing on the short-term advantages of most voters in favour of long-term gains for future generations? Under what kind of political structures dare we hope to see such communitarian and enlightened behaviour? Democratic theory and practice have to face up to such issues (Peters 2019).

## Federalism

### Basics of Federal Institutions

We have considered Swiss federalism as an institutional arrangement that has enabled national unity while maintaining cantonal and regional autonomy. This amounts to a first approximation of most existing federations. Duchacek (1985, 42) put it thus: ‘What water is for fish, the federal system is for the territorial communities that desire to manage their affairs independently (near sovereignly) yet within the confines of an all-inclusive national whole’. Federalism is therefore a political answer to provide a common biosphere for segmented parts of a larger population. Yet it is only an answer to the *territorial* segmentation of society, responsive to the cultural autonomy of language, ethnicity, and so on merely to the degree that these cultures overlap with territorial communities. The carp swimming in a school of pike is not protected against being eaten. There is, therefore, a fundamental difference between federalism and plural democracy. While political pluralism also aims at respecting societal diversity and cultural segmentation, it has no connotation for territorial boundaries.

What characterises federations in the universe of nation-states, where we find a large spectrum ranging from unitary systems like that of France to loose confederations or treaty-like federacies (the US–Puerto Rico) and leagues (e.g. the Arab League)? On the basis of his comparative work on federalism, Duchacek (ibid. 44) finds the following six yardsticks to be the most important:

1. Indestructible identity and autonomy of the territorial components;
2. Their residual and significant power;
3. Equal or favourably weighted representation of unequal units;
4. Their decisive participation in amending the constitution;
5. Independent sphere of central authority;
6. Immunity against secession, that is a permanent commitment to build and maintain a federal ‘union’ in contrast to a confederal system which lacks such a commitment.

Commonly, the first five criteria may be realised as part of the constitutional framework. The sixth yardstick, however, tells us that federalism is more than a constitutional tool used to divide up governmental powers. It refers to the political culture and indeed the political will of a society to constitute and remain a single nation or state. Secessions of the Yugoslav regions and the republics of the Soviet Union show that this political commitment can evaporate if a central government loses its control over centrifugal forces.

Federalism is thus usually adopted by societies where territorial segmentation has led to a political division between forces preferring either centralisation or decentralisation. All federations practise different forms and degrees of shared rule and self-rule (Watts 2008, 35ff.; Hooghe et al. 2016). But this definition is not as clear and distinctive as it seems. A first ambiguity lies in the very word ‘federalism’, which is sometimes associated with ‘ centralisation’, as in Anglo-American parlance, but sometimes a password for decentralising forces, as in Germany or Switzerland. However, this is not just a question of semantics—federalism itself is fundamentally ambiguous. When at least two territorial entities create a new, common government, they give up part of their sovereignty. This process is not only unifying but also centralising. Once the central government is created, the problem of living federalism may well be to guarantee the territorial autonomy of the components, their differences and therefore their relative independence from each other. As Elazar (1985, 23) put it: ‘Federalizing does involve both the creation and maintenance of unity and the diffusion of power in the name of diversity’.

Amongst the 193 member states of the UN, some 25 are known as federations, representing about 40% of the world’s population. We find many other countries which have strong regional authorities, governments and even elected parliaments, such as Italy, Japan, Columbia, France, Peru, the UK (Anderson 2008; Hooghe et al. 2016). Despite considerable devolution of powers and autonomy of the regional governments, these states are not federations but unitary states that—for different reasons—have undergone a process of decentralisation.^4^ What is the difference between a federation and a decentralised unitary state? Looking at Duchacek’s definitions, we find that decentralised unitary states may well meet yardsticks no. 1, 2, 5 and 6. The decisive difference lies in yardsticks no. 3 and 4: only federations let sub-national units participate substantially in national affairs to the extent of amending the constitution ( shared rule), and this under the rule of a favourably weighted representation of unequal units (‘one region, one vote’).

### Structure, Processes and Political Culture

So far, we have considered federalism mainly as an institutional structure, or even as a constitutional framework. Scholars comparing different federal systems all over the world found this institutional scheme useful. But there are limits: ‘Many polities with federal structures were not at all federal in practice—the structures masked a centralised concentration of power that stood in direct contradiction to the federal principle’ (Elazar 1985, 22).

Evidently federalism can be ‘ strong’ or ‘ weak’, and it is more than a structure. Besides varying structural types of shared rule and self-rule, the political process, too, can be federal to different degrees: a strong veto power of sub-national units leads to processes of co-decision in which the central government must respect sub-national interests also in its own fields of competency. Conversely, weak fiscal powers of sub-national units can lead to financial dependency and processes in which the central government controls the use of resources despite formal regional autonomy. Different equilibria of power imply a different appropriate behaviour, which may crystallise into political cultures, too: high veto power of sub-national units favours power-sharing, negotiations on a par and respectful dealing with sub-national units from the side of the central government. In the opposite case, processes between the central government and sub-national units are characterised by hierarchic subordination and majority rule.

**Fig. 6.1:**
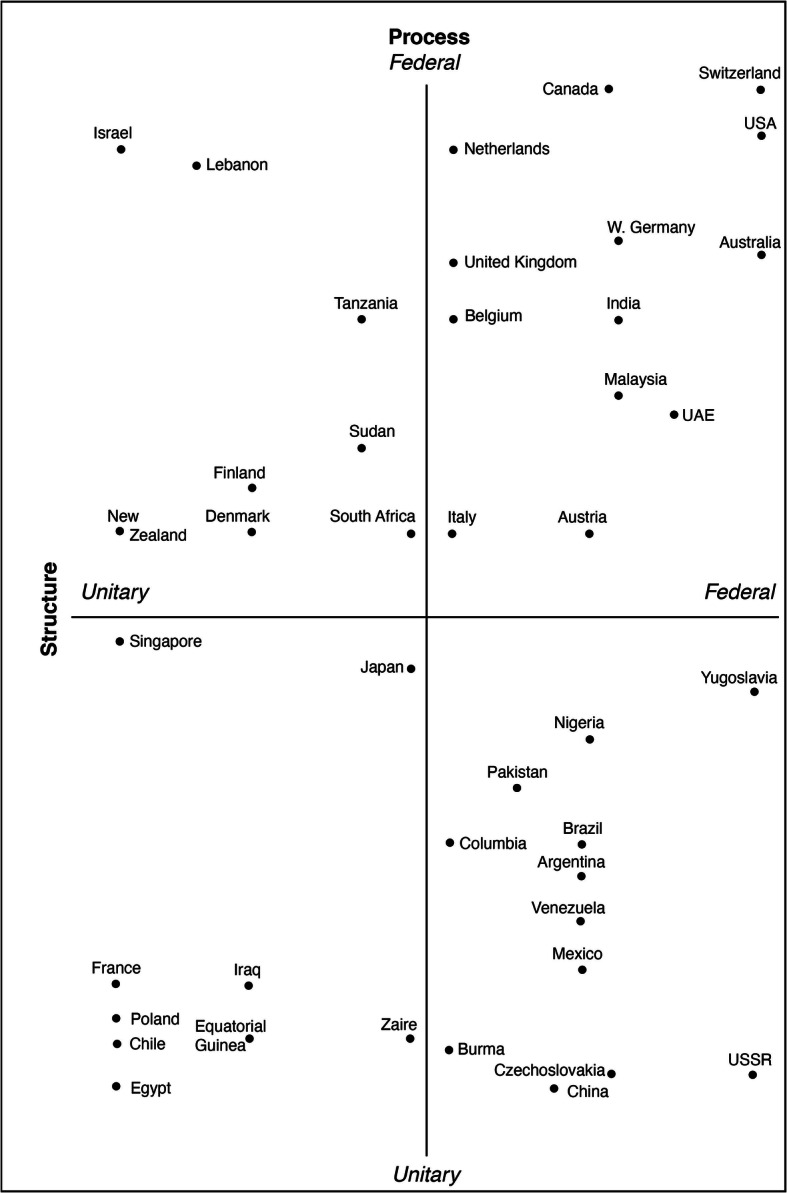
Structure and process in selected polities. (Source: Elazar 1985)

Figure 6.1 illustrates the position of a series of countries on two of these dimensions, constitutional structure and political process. The spectrum ranges from the most federal (upper right) to the most unitary systems (lower left). It presents the situation of the 1980s and is a historical document of the time before the breakdown of the Soviet Union. It shows that some elements of federalism can be found not only in liberal democracies but also in authoritarian regimes. Moreover, the document helps to understand the nature of federalism under a strong central authority: the institutional structures of former Yugoslavia and the Soviet Union were federalist, but central governments monopolised all decisions over resources, controlling the economic activities by way of highly centralised government planning. Meanwhile, the Soviet Union and Yugoslavia ended in implosion or civil war or both. Whereas the extreme concentration of power in these one-party regimes was well known, most observers underestimated the fact that their centralised power also kept together different territorial units with different histories and cultures—artificially, we may say in retrospect, but under structures that were as ‘federal’ in name and structure as those of liberal democracies.

The US and Switzerland are similar cases, being federal in both structure and process. These two oldest federations developed by a bottom-up process, with sub-national units keeping much of their ‘sovereign’ rights as formerly independent states. The veto power of the sub-national units is high, especially in Switzerland where subsidiarity can ally with direct democracy (Mueller 2020). We have already seen that the cantons possess a high financial autonomy and are mandated with the implementation of federal policies (also Vatter 2018). Federal law-making is accompanied by a process of consultation with the cantons. If their reaction to a proposed bill is negative, the federal authorities drop the project or modify it until a solution satisfactory to the cantons is found.

And although the Federal Supreme Court has extensive constitutional power to review cantonal and local legislation, it is reluctant to intervene if sub-national autonomy would thereby be restricted. The federal authorities often do not exercise all the powers they have and, when dealing with the cantons and communes, use their competences with caution. Instead of deciding unilaterally, federal authorities negotiate and respect the cantons or communes as equivalent partners. These non-hierarchical procedures also stem from the need to cooperate. The process of accommodation by the federal authorities of the sub-national units is an appropriate behaviour to find solutions under the conditions of the cantons’ high veto power . It has become an element of political culture, mostly informal, and just occasionally prescribed as a legal procedure. Intergovernmental structures have further stimulated horizontal accommodation among the cantons; they do not compete as much with each other as they could, for instance regarding taxes (Gilardi and Wasserfallen 2016; Wasserfallen 2015).

Taking the two dimensions of process and structure into consideration provides a preliminary picture of the variety of federations and decentralised polities. Elazar’s comparative work showed that there are additional dimensions—such as the coincidence of social and political unity and diversity—which can further describe and explain the operation of federalism. This coincides with the observation of cultural differences that exist between the US and Switzerland, even though both figure at the high end of federalism with regard to structure and processes. Already Chap. 10.1007/978-3-030-63266-3_3 mentioned that Swiss federalism aims at creating equal opportunities in all regions and at equalising policies among the municipalities. US federalism, in turn, stresses territorial competition of state governments, which gives citizens the choice of ‘voting with the feet’. Another cultural difference can be found in the fact that Swiss federalism was conceived to protect territorially entrenched cultural minorities—US federalism was not. This explains why there is not a single federal model, but a rich variety of different types. They depend not only on political structures and processes but also on the history, the specific political culture and the socio-economic challenges and cleavages present in a polity.

### Modern Meanings of Federalism

#### Cultural Autonomy and Difference

The case of Switzerland is instructive for the realisation of political unity whilst maintaining cultural diversity : the 26 cantons, with their different traditions, histories, languages and religions, most of them having enjoyed centuries of political autonomy, were able to create a modern territorial state as early as in 1848. Without federalism and its principle of dividing power between the new central government and the ‘old’ cantonal authorities, and without the federal promise to maintain and even safeguard regional differences, this historical process of the nineteenth century would not have resulted in successful nation-building.

Meanwhile, religious differences have faded. And even if we can still distinguish German-, French- and Italian-speaking cantons, the language boundaries, which never coincided entirely with cantonal ones, have been penetrated by print, electronic and social media and thus become more fluid. Switzerland today is a comparatively homogeneous society. But the Swiss would never contemplate giving up federalism. Despite complaints about federal particularities that may sometimes become obsolete or troublesome, the Swiss like the formal autonomy of their 26 cantons and ca. 2200 municipalities, which in many respects may be fictive and appear to the foreign observer as an institutional luxury in a country of only 8.6 million inhabitants.

Bottom-up state-building and the (con)federal experience are a historical legacy that has shaped a strong preference for ‘small government’ up to our days and helped to develop the idea of subsidiarity: central government should not meddle in things that the cantons are capable of doing themselves, and the cantons should not bother with problems that the municipalities can handle. However, subsidiarity can lead to too small solutions, because the lowest federal level defines *what* the problem is. If the refusal of necessary centralisation is sometimes deplored, it offers opportunities for living ‘differently’. Decentralised trial-and-error processes allow for political innovation, and successful experience can be transferred to upper levels in the sense of ‘best practice’.

Political institutions are not only rooted in and adapted to specific cultural needs, they are part of the social culture. Some say the Swiss feel Swiss only when abroad—when at home they are *Genevois*, *Thurgauer* or *Ticinesi*. Nationalism in the sense of exaggerated pride in the one and only, the chosen people, its language and superiority is thus not possible: between regional cultures and awareness of four linguistic groups, the Swiss are part of a greater, international culture of French-, German- and Italian-speakers. Thus, the Swiss federal structures have remained intact, even though many of their original rationales have disappeared over the last 170 years.

Are these connotations of a federal structure and its associated way of living just a styled reminiscence of the past or are they meaningful also beyond the case of Switzerland and in today’s world? The following provides some answers by illustrating a few of the many facets of federalism.

#### Federalism in Times of Globalisation

Today, the nation-state seems to be too small to handle problems of national security and climate change, to guarantee human rights or to find answers with respect to growing inequalities between industrially advanced and developing countries. With globalisation, international organisations have multiplied, and nation-states have transferred more and more powers to the inter and supranational level. To some extent, supranational organisations resemble ideas of federalism: they decide certain affairs by majority but respect Duchacek’s yardstick no. 3 of ‘equally or favourably weighed representation of unequal members’.

In the UN General Assembly, for instance, China, Liechtenstein and Switzerland are represented equally, each by one single seat. This gives small countries an over-proportional influence on decisions. But we also see that this advantage should not be overestimated: five big powers are permanent members of the Security Council and each has a veto. Closer to the ideas of federalism comes the EU. Besides favourably weighed representation of its unequal members in most of its institutions, the EU Commission, EU Parliament and the Council of Ministers allow members to influence decisions in different ways, and on matters requiring unanimity every member has the right to veto the decision. We may say, therefore, that the development of the international community is to a lesser or stronger degree influenced by structural and procedural ideas of federalism. This is part of the solution to the problem of the nation-state having become too small.

Globalisation and internalisation, however, are contested on different grounds: that they widen inequalities between the first and the third world, that global capitalism tramples on the environment, that policies of the international community lack democratic legitimacy or destroy national structures and cultural identities—including the state itself, which in the high times of neoliberalism was often reduced to a ‘minimal state’. After the financial crisis of 2008/2009, however, the state had to intervene as ‘last resort’ in order to save the whole economy from a total collapse. All this could lead to a re-affirmation of the role of the nation-state—all the more so since despite worldwide capitalism the redistribution of wealth ( social security) and the production of important collective goods (education and health) are still undertaken by the nation-state. Nothing showed this clearer than the coronavirus pandemic in 2020, with states suddenly outbidding each other for essential equipment.

If the nation-state is brought back in, what will be its future structure? While some scholars doubt that federalism can survive in a globalised world, others see modest impacts or even countervailing developments (e.g. Kelemen 2002). Indeed, in many European countries and beyond we observe some important and long lasting trends (Hooghe et al. 2016; Ladner et al. 2019): decentralisation, the rising salience of local and regional politics, social and political movements claiming greater territorial autonomy and the growing awareness of linguistic, ethnic or cultural minorities to defend their identity and to claim new, collective rights. For the protagonists of all these phenomena, the state is not too small but rather *too big*, incapable of dealing with societal diversity at nation-state level. Decentralisation or even federalisation are institutional answers to that problem. Spain, the UK and Belgium, once unitary-centralised systems, are examples where regionalisation took place in reaction to claims for greater regional autonomy. Others may follow.

#### Federalism in Developing Countries

The process of international development and modernisation is, in the first instance, a clash between the worldwide penetration by capitalist enterprises seeking new markets, on the one hand, and self-sufficient local economies and cultures, on the other. In many developing countries, the structures of government that ought to mediate this encounter have not found solutions for dealing with the inevitably arising conflicts. Above all, young democratic regimes, often seduced by short-term gains of centralisation or a charismatic concentration of power, fail to combine selective economic modernisation with targeted backing of indigenous traditions and cultures.

There are structural reasons for this. Many states were created by colonial powers, artificially uniting different ethnicities under one common roof, a problem returned to in the next paragraph. Countries in sub-Saharan Africa, in contrast to many in Asia, lack the cultural heritage of a state overarching familial and clan structures (e.g. Wimmer 2018). Top-down state- and nation-building after the end of European colonisation was a moderate success: central governments not penetrating their peripheries, abuse of political power and widespread corruption are keywords associated with the phenomenon of unsuccessful or even ‘ failed states’. Failed states, however, may be the wrong term and just an episode. European countries needed centuries for their nation-building and were not exposed to the global stress of ever faster socio-economic modernisation. Seeking to improve the political structures of developing countries in the long run, decentralisation and federalisation have become important concepts for developing agencies (Kälin 1999; Litvack et al. 1998; Linder 2002).

 Decentralisation is said to bring the state ‘closer to the people’, giving them a better voice for their needs. But overcoming clientelism and clan politics is possible only if a ‘neutral’, non-familial institution like the state is trusted by citizens. People have to learn that public goods are not gifts from a Big Man but the return of their own fiscal contribution. And they must have the confidence that this return will be fair, effective and corresponding to their needs, which implies learning processes also for the political elites. Local autonomy, fiscal decentralisation or even federalism can increase the chances for this learning process to occur compared to unitary-centralised government (Oluvu and Wunsch 2004; Linder 2009). They represent a promising alternative to the mainstream politics of post-colonial period, namely bottom-up state- and nation-building .

#### Federalism as a Guarantee for Cultural Difference and Diversity

While federalism in Belgium, Switzerland and Canada serves to unite the diversity of only a small number of cultural groups, Nigeria or India are much more complex. In these cases, federalism must unite the cultural diversity of dozens of ethnic groups or hundreds of different languages. Thus, federalism is sometimes used as a synonym of the guarantee for cultural difference and diversity, regardless of history or socio-economic circumstances. But is this true, and to what degree can cultural minorities be effectively protected?

First, we have to note that not all federations were designed to ensure cultural diversity . Indigenous peoples in the US, for instance, are protected through reservation areas but do not benefit from political autonomy in the form of their own state. As a nation of immigrants, the US still favours the ‘melting pot’ concept: it trusts the idea that the dominating white, Anglo-Saxon and Protestant culture will assimilate all immigrants. The more important question, however, is whether federalism is really capable of protecting cultural difference and diversity, if that is the goal.

The experience is mixed. In South Africa, federalism seems to play an important role for the consolidation of a deeply divided society (Lemarchand 1997). But under the common roof of India’s or Nigeria’s immense cultural diversity , some shadows exist: there is evidence that in situations of serious crisis, federal structures in both countries are not used to solve conflicts (Iff 2009). In Canada, federalism could not prevent the French-speaking province of Quebec from twice calling a plebiscite on independence, in 1980 and 1995. In Belgium, which grants its two segments of French- and Dutch-speakers the utmost autonomy, national unity is said to be fading (Deschouwer 2012), held together only just by common symbols such as the monarchy, football, chocolate and beer.

This reminds us that federalism, giving either too little or too much way to minorities, runs the double risk of paving the way for unitary systems or breaking apart. One should not confound effects and cause, however. Modest success is partly due to the fact that it is primarily divided societies trying to integrate minorities through federalism (e.g. Walsh 2018). Such is the case with the most recent projects of federalisation in Nepal, Myanmar or Syria.

It may be useful to look at both the potential and limits of minority protection from a theoretical perspective (Kälin 1997; Linder 1997). The following conditions seem pertinent:

1. *Minorities* *not too small in* *size* *but sufficient in number*: Evidently a 20% minority has greater chances to benefit from federal autonomy than a minority of 2%. For a single minority group, however, size alone may be of little help: it is always the same (regional) conflict which is at stake, and despite federalism, the same majority will have the last word. Conflicts may accumulate, as for instance in the former Czechoslovakia, which dissolved in 1993. If instead regional autonomy is claimed by several and different kinds of minorities, chances of protection are better: the problem becomes more ‘objective’, coalitions change, and compensations between different actors and issues are possible. Too great a number of minorities divided up into many units, however, may become a disadvantage. Nigeria, for instance, started with three regions, in 1960; today, not less than 36 ethnic groups each have their own territory. While this may be reasonable from the point of view of a single ethnic group, it lessens the influence of sub-national units over the central government, which can resort to a strategy of ‘divide and rule’.
2. *Cross-cutting* *cleavages*: A single region may be characterised by several political characteristics, for example, belonging to both a religious and linguistic minority whilst also being relatively poor. In this case, conflicts accumulate, as we have seen in the case of the Jura region, whose predominantly Catholic and French-speaking population also felt neglected economically by Protestant, German-speaking Bern (see Chap. 10.1007/978-3-030-63266-3_3), and chances of minority protection are less propitious than in situations of cross-cutting cleavages. If a minority region is not poorer but wealthier than others—as for instance the Basque Country in Spain—chances of its autonomy being respected are much more favourable.
3. *Effective political majority in a sub-national unit*: Federalism only protects territorially segmented minorities, as in a pond which is divided into two parts, one for pikes and the other carp. But a carp swimming in the pikes’ part is not protected against being eaten. Similarly, even a large minority cannot benefit from federalism if does not constitute a political majority within the boundaries of at least one sub-national unit. For example, in Switzerland Muslims exceed the population of an average Swiss canton but are dispersed all over.
4. *No complete geographical division of ethno-cultural groups along the borders of sub-national units*: In situations of serious conflict, federalisation is sometimes used to separate hostile ethnic groups. This was the case in Bosnia-Herzegovina, when the Dayton Agreement of 1995 drew the borders of the sub-national units along the geographical borders of the Bosniak, Serb and Croatian communities. This helped foster peace at that time but inadvertently continued the policy of ‘ethnic cleansing’. It led to ethnic regions with the risk of creating their own, internal minority problems. With ethnic political parties, the ethnic cleavage and its conflicts may remain the central concern of all politics. To a certain extent, this point thus seems to contradict point no. 3: minorities should be able to constitute a majority in, but not be able to exclusively dominate, a sub-national unit. Yet, this is not a contradiction, rather an unresolvable paradox: every minority protection through federalism creates a new minority problem. After each opening of a Russian nested doll, a smaller Matryoshka becomes the biggest one. Under inversed roles, the minority in a country having become the majority of a sub-national unit has to find a new way to protect its own minority.

Looking at these four points, we notice that in Switzerland minority protection has benefitted from favourable conditions: the number and size of minorities was neither too small nor too large. Religious, cultural and economic cleavages were cross-cutting; this facilitated the development of national political parties which are not confined to language or ethnicity. Cross-cutting cleavages had the side effect that every member of the political elite is somewhat part of a minority *and* a majority. A Radical, Catholic and French-speaking candidate from Valais has the advantage of belonging to the linguistic and religious majority of her canton, but the handicap of politically representing a minority in a Christian-Democratic stronghold. Once elected to the National Council, however, she belongs to the bourgeois majority but the linguistic minority. Being in the majority and the minority at the same time is the experience of most Swiss politicians and citizens.

If these favourable conditions have aided a successful dealing with minority problems, it should be noted that federalism alone would probably not have helped much in the Swiss case. Federalism is only one part of the solution for minority integration, in Switzerland as much as elsewhere. To achieve minority protection, federalism must be embedded in other institutional devices such as a non-religious, non-ethnic concept of the state, a strong and effective tradition of human rights and institutional elements of political power-sharing (Fleiner et al. 2003).

#### Federalism and Democracy

Democracy is basically majority rule founded on the number of votes cast, each voter having an equal weight , whereas federalism implies equal or favourably weighted representation of uneven units. A common pattern of institutionally combining the two modes is bicameralism : government proposals have to be voted on in two parliamentary chambers, one representing the people, the other the member states. Yet, there are many ways to proceed. While taking part in the deliberation of all federal laws, Germany’s *Bundesrat* has full decision-making powers only in matters with consequences for the *Länder*. The chamber itself is composed of government representatives of the member states. Switzerland requires double majorities in parliament and a popular vote for any amendment to the Constitution, whereas the ratification of amendments to the US Constitution proposed by two-thirds majorities of Congress relies on individually organised procedures of the states, where a majority of three quarters is required. In all these cases important government proposals have to find a double—or ‘compound’—majority.

Inevitably, the federal protection of territorial groups leads to a distortion of the democratic principle of equal representation. The votes of individuals or representatives of member states with a small population are weighted more heavily than those of large member states. They can organise a veto to block democratic majorities. For Switzerland, where cantonal population size varies at a ratio of 1:42, we have already discussed the implications of the theoretical veto power of the smallest member states, who represent just 21% of the population (see Chap. 10.1007/978-3-030-63266-3_3). In other countries, such as the US, with similar population differences between its units, the consequences may be less important because a divide between large and small states is unlikely to happen. But there is no doubt that federalism, with its compound majorities, implies an infringement on the democratic principle of equally weighted votes (see also Mueller 2020).

Federalism has, however, two main advantages that can compensate for this cost. First, when conflicts arise, federalism is a constraint that ‘forces’ democratic majorities to bargain with federal minorities . In general, this favours the status quo. In practice, however, the reverse has applied in Switzerland too. Minorities of cantons may introduce innovations within their boundaries for which majorities at the national level are not found. Later, when the innovation proves successful at cantonal level, the innovation is accepted throughout. Federalism is therefore not only an institution ‘forcing’ negotiation to take place, but one that provides opportunities for social learning by trial, error and innovation.^5^

Second, the democratic costs of federalism at national level can be compensated for by democratic gains in the regions. In fact, democratic federations are mostly conceived as multi-level democrac*ies* whose constitutions prescribe the same standards of liberal democracy for member states and local governments. In such multi-level democracies, the political rights of citizens—the election of government officials, parliamentary members and so on—have a much greater significance. Not only can voters participate more often, but they can also vote for different parties and persons at different levels. A voter can express different preferences in local, regional and national politics. The frequency of elections provides citizens as well as authorities with permanent information on the popularity of ruling majorities. This phenomenon can be particularly well observed in Germany, where 16 *Länder* governments are elected during one term of the federal government. Changes of power in parliamentary democracies often make their way up and down the federal escalator. In a federation, not only the state but also democracy is closer to the people.

#### The Question of Secession

At a congress of East-European and Swiss constitutional lawyers held in Lausanne in 1990, one unforeseen issue dominated the discussions: how may a canton secede from the Swiss federation? Participants from Lithuania, Ukraine, Croatia and other places, eager to obtain advice on the then emerging desire for national independence, were somewhat disappointed to hear that neither the Swiss Constitution nor legal scholars had thought much about the question of secession. Meanwhile, the history of Yugoslavia has given a series of answers: the de jure recognition of the first de facto secession of Croatia through West-European countries, the breakdown of the federation in an atrocious and destructive civil war, the secession of what is now North Macedonia by popular vote and, in the case of Kosovo, again the de jure recognition of a de facto secession by several other countries and the international community. While civil war is to be rejected without discussion, the other answers leave many doubts. Should federations regulate secession? Can we think of a ‘right’ to secede, and if so, what should be the procedure and what would be its consequences?

International law provides only some general, fragmentary answers (Thürer and Burri 2009). Secession is lawful under the narrow circumstances of severe violation of human rights and in cases of de-colonisation. Nothing is said about federations and their paradoxical particularity: the federal polity gives its components ‘indestructible identity and autonomy’, which makes it more vulnerable to secession. At the same time, a federation is conceived as a permanent union—in contradistinction to a confederal system which lacks such commitment. From this perspective, a secession clause seems to be needless: federalism, in historical perspective, is successful when it transforms a constitutional arrangement into a commitment felt and accepted by all regions and their citizens, thus rendering the question of secession obsolete.

But this historical process can fail. Cultural segments may recall ancient dreams of independence well beyond federal autonomy. There may be territorial segments that are systematically discriminated against. Instead of shaping the collective memory of a respectful pluralist experience, the passing of time then provides undeniable ‘proof’ of discrimination, creating alienation and justifying hatred among different groups (Esman 1990, 14). Behind many ethnic conflicts we find the economic question of redistribution. One region is unwilling to share the wealth coming from its natural resources with others, or inequalities of productivity and wealth are growing instead of diminishing. Another part of the country may feel to be the permanent loser. Conflicts on questions of the economy, language, religion and culture may escalate and end up in deep divides. Once secession becomes unavoidable, a ‘peaceful divorce’ like the one in Czechoslovakia, where both parts in 1992 agreed to go separate ways, is unfortunately the rare exception. Rather we find a territorial minority seeking self-determination and secession against a majority of citizens who find it justified—and may even demand—that their national government defends the integrity of the state.

The case of Catalonia is highly instructive in this regard: as a reaction to the growing assertiveness of Catalan independentists in the 2010s, the far-right party VOX, which aims to defend Spanish unity and integrity, became suddenly very popular. In the name of national unity, the conservative government of Spain was intransigent, even oppressive against the regional movement and its political leaders. As it refused to propose procedures designed to bring about a peaceful solution, the conflict is not overcome but stalled. The left-wing government of Pedro Sánchez, installed in January 2020, has at least agreed to a dialogue with the Catalan regional government.^6^ But at the same time it must be careful not to lose its political support in the rest of the country.

Thus, it may not be absurd to formulate future secession rules. Two questions would have to be answered. First, under what *circumstances* should a federation be obliged to let one of its members go? If any member is able to quit any time, the federation cannot function. If the decision has to be made unanimously by all members, the rules may be irrelevant because secession may become impossible. Therefore, the answer must lie somewhere in between. Second, *who* should have the right to claim secession? This question may be crucial because within the boundaries of a secessionist member state, we may find a (large) minority who would like to stay within the federation.

The case of the Jura region separating from the canton of Bern is instructive in this regard. As described in Chap. 10.1007/978-3-030-63266-3_3, first the people of every district and then also of every border municipality were given the right to decide on whether to stay with Bern or secede into the new canton of Jura. Thus, it was the popular majority in each district or even commune that defined the territorial boundaries of secession. The region was cut in two—one remaining with the old canton, the other founding its own. Although some political forces on both sides of the new border ended up unhappy, the division at least prevented the creation of a new minority problem: the minority that wanted to stay with the old canton was not overruled and was given the same right to self-determination as the separatist majority. Yet even here, fragments of the conflict linger on: in 2017, the city of Moutier voted anew and decided to join Jura, but the result was later cancelled by the courts because of anomalies during the campaign and voting process. At the time of writing, when the vote will be repeated is unclear.

This leads us to the following conclusion: in most cases, territorial secession gives rise to as many new minority problems as it claims to resolve. This is inevitable where territorial segmentation is not perfect. In the Czechoslovak ‘divorce’, for instance, the Slovak minority wished to free itself of Czech majority rule. But on Slovak territory today we find a minority of about 9% Hungarians among the 5.5 million Slovaks, as well as other important minorities such as Romani, Czechs, Russians, Ukrainians, Romanians, and so on—we are reminded again of the matryoshka allegory mentioned above. Therefore, the once popular idea of a nation-state based on one language or culture—still claimed by many secessionist movements—is ill-founded.

International law may inadvertently promote this problematic idea. The right to a ‘people’s self-determination’ is increasingly used as an argument for secession also by ethnic groups. The difficulties in defining the ‘people’ that should be granted ‘ self-determination’ may lead to inconsistent interpretation and opportunistic intervention by the international community. In this respect, a comparative look at the secession of Serbia’s Kosovo, Georgia’s Abkhazia and South-Ossetia and Ukraine’s Crimea is revealing (Hehir 2009, Nielsen 2009, Paech 2019, 93).

Federations, all other things being equal, are more vulnerable to secession than unitary states. Two policies may help safeguarding their unity: one, to find solutions other than secession; two, to find these solutions without interference from the outside. As to the first policy, giving problematic regions special autonomy status is a reasonable alternative to eventual secession. It is a compromise that may ease tensions and leave both parts better off, as with Spain’s Basque Country. Special arrangements with particular sub-national units are known as ‘asymmetric federalism’ in the constitutions of India, Malaysia, Belgium, Canada and others (Brown 2005; Watts 2008).

Second, rules for secession should serve the one and only objective of preventing future secession. This seems paradoxical at first but is not. Rules of secession may change the balance of power: openly and clearly specifying the conditions of eventual secession may strengthen the position of sensitive territorial minorities and give them more bargaining power against the central government. If installed well before a conflict breaks out, such rules may lead to more cooperative processes in the federal polity and reduce the risk of secession. Two young federations, both with considerable potentials of conflict, Ethiopia and Sudan, have installed rules for secession. While in the latter case the South seceded in 2011, time will tell whether in the former the provision works as proposed here.

### Non-Territorial Federalism

The idea of a territorial state that has exclusive power over all the people living within its borders is relatively recent. The older concept of political power was based more on the idea of personality. For instance, following the Germanic invasions of various provinces of the Roman Empire, there lived—side by side and under the sway of the same ‘barbarian’ ruler—ex-Roman citizens and members of one of the Germanic tribal confederations (such as Goths, Vandals, Burgundians, Franks and Lombards). Yet in most cases, and over a considerable period of time, the two groups remained distinct entities, and what mattered before the law was *who* the defendant was, not where he was living. Romans were judged by Roman law, the new Germanic settlers by their old Germanic customary law. Both groups regarded this practice as proper and, indeed, as ‘a precious safeguard of their respective rights and privileges’ (Ra’anan 1990, 14).

With industrialisation and the development of bureaucratic statehood, West-European countries led the way in becoming territorial states. Under the principle of *ius soli*, the territorial state claims full jurisdiction over its citizens—whatever their origin. Earlier we described part of this evolution for Switzerland. In its religiously segmented society of the nineteenth century, marriage and education were regulated and organised separately for Protestants and Catholics by their churches. Whereas the label ‘State Church’ has not completely disappeared, churches have by now mostly lost their status as actors in public affairs in favour of the confessionally indifferent state which provides for Protestant and Catholic citizens alike and under the same laws.

Yet, the principle of *ius sanguinis* has not completely disappeared. In the last days of the Austro-Hungarian Empire, Karl Renner and Otto Bauer proposed forms of non-territorial or corporate federalism to resolve the nationalities problem: ‘Within each region of self-government, the national minorities shall form corporate entities with public judicial status, enjoying full autonomy in caring for the education of the national minority concerned, as well as in extending legal assistance to their co-nationals vis-à-vis the bureaucracy and the courts’ (cit. in Ra’anan 1990). Such corporate federalism was introduced for cultural minorities in Estonia in 1925, in Cyprus under the 1960 Constitution and lately for Burmese minorities (Coakley 2017).

The most prominent example, however, is Belgium where federalisation since 1970 has taken both territorial and non-territorial forms. The country is divided into the regions of Flanders, Wallonia and Brussels. But Belgium is also divided into a Flemish- (comprising both the territorially defined area of Flanders and the corporately defined group of Flemish-speakers in Brussels), a French- (comprising both the region of Wallonia and francophone *Bruxellois*) and a German-speaking community (Eupen/Malmédy located within Wallonia) (Jans 2000; Deschouwer 2012).

Corporate federalism allows a minority to maintain its own public institutions without territorial segmentation. This raises two questions. The first is: what are the limits of cultural minorities’ right to run their own public institutions? This eventually depends on the concept of the state, the constitution and a society’s ideas of pluralism. Therefore, we find different answers even for the same issue. In Switzerland’s public education, for instance, French-speaking schools in the German part of the country are well accepted as an element of multilingualism . Religious schools, however, were declared non-constitutional by the laic majority of the nineteenth century because in its view these schools violated the separation of state and church. Today, schools of religious and other communities are tolerated under certain conditions but at any rate must respect constitutional freedoms, such as gender equality or freedom of speech. Constitutional law sets the principles which are to be respected by all segments of a pluralist society. But these principles and concepts of pluralism vary considerably.

The second question deals with consequences: can non-territorial federalism keep the balance of unity and diversity, or do parallel institutions, exclusively reserved to cultural minorities, lead to ever deeper social divisions undermining unity? In the literature, the question remains controversial. While some observers of the Belgian case fear the latter, others see non-territorial federalism as a promising approach to ‘identity politics’ (White 2000; Deschouwer 2012).

## Power-Sharing and Consensus Democracy

### Majoritarian and Consensus Democracy: A Comparison

If there is one continuous thread in Swiss political history, it is probably the desire to prevent winners from taking all, leaving losers with nothing—or, in other words, power-sharing. It is found in the Constitution, in the federal bargain between Protestants and Catholics, in the compromise between centralists and partisans of cantonal autonomy, and in the development of proportional representation—first for the election of parliament, then for the Federal Council, and later for the bureaucracy, expert committees and even the courts. All this gives minorities the opportunity to participate. The law-making political elites, in order to minimise referenda risks, try to arrive at a political compromise that includes all important political groups. Power-sharing provided the solution to the problem of integrating a heterogeneous, multicultural society by political means. It has led to a type of democracy different from others.

The combination of these elements through the Swiss *Konkordanz* , which avoids alternating government and opposition forces, may be unique but power-sharing, as a mode of democracy different from majority rule, is not. Arend Lijphart (1969, 1977, 1984, 1999, 2012), a prominent scholar comparing political institutions, has called this ‘consociational’, ‘power-sharing’ or ‘consensus’ democracy, a type of democracy different from the ‘majoritarian’ or ‘Westminster’ model of democracy (Table 6.2).^7^

**Table 6.2:** Lijphart’s types of majoritarian and consensus democracy

	Majoritarian democracy	Consensus democracy
1. *Executive*	Concentration of power in one-party and bare-majority cabinet	Power-sharing in broad coalition cabinet
2. *Relations between government and parliament*	Cabinet dominance	Balance of power
3. *Political parties*	Two-party system	Multi-party system
4. *Electoral system*	Majoritarian and disproportional	Proportional representation
5. *System of interest groups influence*	Pluralism	Corporatism
6. *Government structure*	Unitary and centralised	Federal and decentralised
7. *Parliament*	Concentration of legislative power in unicameral legislature	Strong bicameralism
8. *Type of Constitution*	Flexibility, simple procedure of amendment or unwritten constitution	Rigidity, complex procedure of amendment
9. *Judicial review*	Absent or weak	Strong
10. *Central bank*	Controlled by executive	High degree of autonomy

Source: Lijphart (2012)

These two types of democracy represent coherent and therefore ideal polities maximising the basic ideas of either majoritarian or power-sharing politics. It is easy to identify Switzerland and the UK as two polities that correspond to most criteria of one of the models. The UK systematically favours the logic of majority rule: competitive elections between two main parties based on one major political division (left-right) lead to clear parliamentary majorities. The winner-takes-all rule makes parliamentary majorities sensitive to even small changes in the electorate’s preferences; the losing party becomes Her Majesty’s Official Opposition. Because of its parliamentary majority, the executive cabinet is empowered to realise its policy programme, as long as there is no successful vote of no confidence, which may necessitate an early election. Power is concentrated among the parliamentary majority and the cabinet. The House of Lords has few competencies; almost all legislative power belongs to the House of Commons. The latter may change constitutional documents in the same way as any other laws, with very few judicial constraints. One may speak of a nearly ‘sovereign’ parliament, with the main exceptions of devolution of power to Scotland, Wales and Northern Ireland , and of some independence given to the Bank of England. A similar coherence of elements, but with the opposite goal of power-sharing and negotiating politics, is found in the consensus model of Switzerland. Lately, both Switzerland and the UK have somewhat moved away from the ideal models in becoming less consensual, the first, and less majoritarian, the latter.

**Fig. 6.2:**
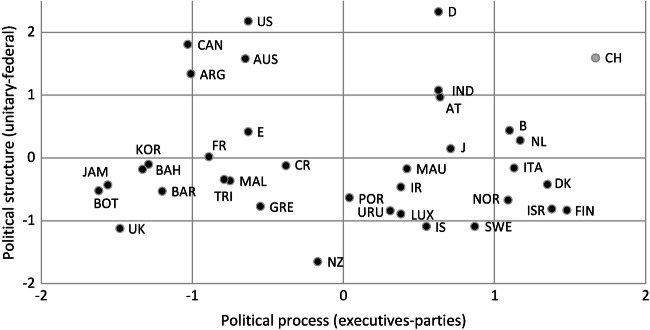
Majoritarian and consensus democracy: a two-dimensional conceptual map. (Source: own calculations and figure, using Lijphart’s (2012) data. Shown are average values for 1981–2010. Countries: *ARG*: Argentina; *AUS*: Australia; *AT*: Austria; *BAH*: Bahamas; *BAR*: Barbados; *B*: Belgium; *BOT*: Botswana; *CAN*: Canada; *CR*: Costa Rica; *DK*: Denmark; *FIN*: Finland; *FR*: France; *D*: Germany; *GRE*: Greece; *IS*: Iceland; *IND*: India; *IR*: Ireland; *ISR*: Israel; *ITA*: Italy; *JAM*: Jamaica; *J*: Japan; *KOR*: South-Korea; *LUX*: Luxembourg; *MAL*: Malta; *MAU*: Mauritius; *NL*: Netherlands; *NOR*: Norway; *NZ*: New Zealand; *POR*: Portugal; *E*: Spain; *SWE*: Sweden; *CH*: Switzerland; *TRI*: Trinidad and Tobago; *UK*: United Kingdom; *URU*: Uruguay; and *US*: United States)

Majoritarian and consensus democracy are more than descriptions of two special cases in abstract terms. Lijphart’s typology was particularly seminal in a comparative perspective. His updated study of 2012 shows how 36 countries can be situated on a continuum from majoritarian to consensus democracy. In this two-dimensional Fig. 6.2, Lijphart’s ten criteria are organised into two groups. The horizontal dimension sums up all indicators of the political process of parliament and government that lead to majoritarian or power-sharing politics (characteristics 1–5 in Table 6.2). On the vertical dimension, we characteristics 5–10, which essentially represent a unitary-federal continuum. Unsurprisingly, almost all federations— Canada, the US, Austria, Germany, India and Switzerland—are located in the upper part.

Federalism therefore shows up as an important structural element of consensus democracy but is not as decisive as one could expect. Canada and the US are two countries combining federalism with majoritarian processes of politics. In the Scandinavian countries, Lijphart found only characteristics of power-sharing unrelated to federalism: multi-party systems, proportional representation, grand coalition cabinets designed to integrate different political forces, corporatism and a balance of power between cabinet and parliament. We note that the UK and Switzerland, as mentioned above, still end up as ‘ideal’ majoritarian or consensual cases because their respective logics of structure and process coincide.

Does power-sharing make a difference? Yes, says Lijphart. In many of his comparative studies he found evidence for a different performance of politics in majoritarian and consensus democracies:

Indeed, the results could hardly be clearer: consensus democracy—on the executives-parties dimension—makes a big and highly favourable difference with regard to almost all of the indicators of democratic quality and with regard to all of the kinder and gentler qualities. (Lijphart 2012, 294)

With regard to the developing world, we could add a further point: power-sharing helps democratisation. Drawing on Lijphart, Linder & Bächtiger et al. (2005) developed a concept of power-sharing applicable also to non-consolidated democracies or even authoritarian regimes. In a comparative study of 62 countries from Africa and Asia, they found that between 1965 and 1995, power-sharing and the cultural element of low familism turned out to be the strongest predictors of democratisation. Economic factors—often viewed as the most important variables shaping democratisation —had only limited effects.

### Democratic Power-Sharing: A Key to Resolving Conflicts in Multicultural Societies

Chapters 10.1007/978-3-030-63266-3_2, 10.1007/978-3-030-63266-3_3, and 10.1007/978-3-030-63266-3_4 of this book illustrate what political power-sharing has done for Switzerland: complementing federalism, it became the key element in integrating a community of two religions and four languages. Later it provided the Swiss with a collective identity strong enough to defend their political independence in periods of war abroad, and it helped to overcome some class struggles. Power-sharing—considered by political scientists as the most appropriate form of democracy for pluralist or segmented societies—has even turned Switzerland into a relatively homogeneous society, in spite of its different languages. From this perspective, the ‘paradigmatic case of political integration’ (Deutsch 1976) of Switzerland has been an undeniable success. Can power-sharing and consensus democracy also be used by other countries facing the problem of multicultural integration (see also Iff and Töpperwien 2008)?

The question is pertinent. The integration of different cultures through political institutions has become an important issue worldwide, at a much larger scale, and with more difficult problems than in the case of Switzerland. We mentioned India with its many hundred languages and idioms; some of Africa’s sub-Saharan states are faced with the challenge of forming conglomerates of dozens of ethnic tribes which never before in history had been united together under a common political regime. In the new order of worldwide liberalisation and open markets, if the money does not go to the poor, the poor will go where the money is. Millions of people are migrating within the Third World or from the Third World to more developed countries (Milanovic 2016).

This has also led to the intertwining and confrontation of different cultures which once had been quite separated. European countries are experiencing growing immigration from overseas. In California or New Mexico, US states with strong immigration , a considerable part of the population are Spanish speakers. They do not identify with the culture of white Anglo-Saxon Protestants, and the melting pot idea of assimilation is fading. Today, a large majority of the countries considered as sovereign states constitute multicultural societies. Yet conflicts between different groups of language, religion or ethnicity are salient in all regions of the world. Historical minority problems in industrialised democracies have not faded away, and in Europe immigration has led to new social tensions.

Gurr (2000) estimated that at the beginning of the millennium, about 275 minority groups from 100 countries, representing one seventh of the world population, were politically endangered. Instead of classical war between states, we increasingly find armed conflict between different groups in deeply divided societies—such as in Syria, Libya, Sri Lanka or Pakistan, to mention just a few. In many cases, the causes of internal conflict boil down to conflict over resources, but political escalation, alienation and mass mobilisation are often based on cultural difference or intertwined with discrimination (Lake and Rothchild 1998).

In order to prevent minority problems becoming salient or even escalating into violent forms of ethno-politics, more, or better, political integration is needed. Is power-sharing or consensus democracy appropriate for the problems of multicultural coexistence and integration, and if so why?

To begin with, we notice that the predominant model of democracy is majoritarian. Before spreading all over the world, majoritarian democracy was invented and first practised by white Anglo-Saxon Protestants. They shared common cultural values and beliefs and spoke a common language. Westminster democracy is (or was, after Brexit) a perfectly adequate decision-making procedure for the solution of social conflicts in Britain’s industrial society. Part of the voters, not being tied to an ideological position, are open to the question of whether the country needs more liberties for entrepreneurs or more social protection of workers. According to the economic situation and the performance of the last government, the British may vote in a pragmatic way: first for the Conservatives, twice for Labour and eventually again for the Conservatives. This change of individual preferences sums up to changing political majorities and to alternating roles of government and opposition.

In multicultural societies, however, majoritarian democracy may encounter serious difficulties. Cultural values, beliefs and languages are not only heterogeneous, but may also lead to different political preferences that do *not* change: parents cannot opt out of sending their children to schools held in their own language, or discard their religious beliefs, without giving up part of their cultural identity. Individuals or groups cannot ‘free’ themselves from their cultural heritage, or only at great cost to themselves. In such situations, minorities cannot hope to gain much from majoritarian democracy . If the dominant cultural majority is large enough, it will not have to take into account the preferences of the minority (e.g. O’Leary 2019, 558).

In the worst case, a government’s chances of re-election under the winner-takes-all rule even increase if it offers special benefits to its own cultural group while discriminating against the minority. If majoritarian democracy does not offer a regular change of power, it suffers from three deficiencies:

1. Despite elections, the political majority becomes ‘eternal’, which goes against the basic idea of majoritarian democracy .
2. Such an ‘eternal’ government has no incentives to take into account the needs and preferences of minorities. It can afford not to learn, which is the pathologic use of power.
3. Majority rule may further alienate those cultural segments which find themselves always in a minority position.

Tocqueville’s , Madison’s or J. S. Mill’s criticism of democracy as a ‘tyranny of the majority’ is therefore well founded. This has led to corrective institutions, such as rule of law, basic rights for individuals, federalism or particular autonomy rights for regions and minority groups. A further corrective element is political power-sharing. Lijphart, already in the first versions of his theory, proposed that consensus democracy is better suited than majoritarian institutions for multiculturally segmented societies. The theoretical reason is obvious: consensus democracy gives societal minorities a chance to participate in political power and have a voice in the policies of the government which cannot be overheard. By mutual agreement and compromise, societal divides may be eased or even overcome.

Looking at the classical power-sharing democracies of Switzerland, Belgium or the Netherlands, Lijphart’s proposition makes sense. The case of Northern Ireland , where elements of power-sharing were introduced as part of the peace-process between Protestant Unionists and Catholic Republicans, is at least promising (e.g. McGlinchey 2019). Finally, India shows that elements of informal power-sharing can be useful also in developing countries and under conditions fundamentally different from small European states (see, however, Adeney and Swenden 2019).

On the African continent, we find contradictory experiences (Remond 2015): in South Africa, power-sharing enabled the passage from Apartheid to democracy, allowing the white minority as well as different black ethnicities to participate. The power-sharing pact in Rwanda in 1993, however, could neither outweigh conflicts on resources nor put an end to the historical hostilities between Hutu and Tutsi, once fuelled by the colonial powers. An atrocious civil war followed. Moreover, peace agreements in divided societies such as in Bosnia, Cambodia, Burundi or East Timor, often arranged by the international community, proved to be of moderate success despite provisions for political power-sharing (Mukherjee 2004). Against this background, one is not surprised to find a controversial academic debate. Critics of Lijphart state that power-sharing is not helpful for peace-making or even that it undermines democratisation (e.g. Sisk 1996; Roeder and Rothchild 2005; Norris 2008; Lijphart 2008).

Much of this academic critique departs from an inadequate baseline, as it does not compare majoritarian with consensus democracy as a sustainable institutional arrangement under equal conditions. Rather, it evaluates the short-term success of power-sharing agreements as part of the peace-making process. It is obvious that the transformation of peace-treaties into stable democratic institutions bears high risks and can fail for many reasons. From a vast literature, one can learn that the consolidation of democracy entails a long process also under more favourable conditions than present in war-torn societies. In developing countries, much depends on the existence of a consolidated state, chances for economic development and the compatibility of the cultural heritage with social modernisation (Senghaas 1997; Carothers 1999; Leftwich 1996; Moore 2001; Przeworski et al. 2000; Linder and Bächtiger 2005).

Power-sharing peace arrangements after armed conflicts in deeply divided societies may be a good beginning, but that is not the same as an established constitutional order, and only part of a consensus democracy yet to be developed. When it boils down to the relevant question of comparing majoritarian with power-sharing institutions, empirical evidence favours the latter (Lijphart 2008; Norris 2008):

1. *Proportional* *representation* *has a high symbolic value, favouring the development of mutual respect between different cultural groups*. The self-esteem and political recognition of minority groups are an essential precondition for any rational political discourse and accommodation among elites. To promote this objective, proportional representation can be practised in many places: in the electoral system, in parliament, in the executive, in all branches of the administration or also in the police and armed forces. Of course, proportional representation has some pitfalls. Under the conditions of one single minority or a single cleavage, there is a risk that proportional representation perpetuates societal conflict instead of cooling it down. With more than one minority and cross-cutting cleavages , however, proportionality may favour the development of non-ethnic, non-regional political parties, elites and cultures. The evolution from a divided into a pluralist society lets old cleavages fade into the background.
2. *Proportional* *representation* *favours negotiation and accommodation of conflicts whereby minorities have an effective voice*. The veto power of minorities does not suspend the formal rule of majority decision. Yet, where minorities are permanently participating in decisions, formal decisions imply negotiation and accommodation, avoiding ‘winner takes all’ situations and mindsets. For example, right since 1848, French-speakers have *always* had at least one, most often two representatives in the seven-seat Swiss government (Giudici and Stojanović 2016, 297). The effective voice of minorities depends on two conditions. The first is mutual recognition of the different parts of the political elite. This opens the door to cooperation on a rational basis. On such a basis, solutions turning zero-sum into positive-sum games become feasible. Cooperation then is more advantageous than non-cooperation because it leaves all parts better off. The second condition is alternating, issue-specific coalitions. If today’s opponent is tomorrow’s coalition partner, both are partly dependent on each other. This favours a political culture of mutual respect and support. Empirically, under power-sharing conditions politicians listen more to each other and give more weight to arguments of their opponents than in majoritarian situations (Bächtiger et al. 2005; Steenbergen 2009). Thus, proportional representation and power-sharing are more promising arenas for deliberative democracy.
3. *Political* *cooperation* *among political elites may encourage general patterns of amicable intercultural relations*. Cooperation in parliamentary and executive bodies not only promotes compromises on political issues. It may also, through frequent interaction and mutual dependency, lead to a better understanding between different cultural segments and the development of common values. This process may at first be limited to the political elites, but it can then ‘trickle down’ to larger segments of society.
4. *Federalism or* *decentralisation* *may be more effective for multicultural co-existence if combined with other elements of power-sharing*. Federalism may be considered a structural element of power-sharing. While restricting the power of the central government, it can guarantee autonomy for different cultural segments in territorial sub-divisions. Like basic individual rights or statutory minority rights and vetoes, federalism is an institutional mechanism restricting majority rule and limiting majority politics. Federalism as a ‘vertical’ dimension of power-sharing has its deficiencies, however, as we have discussed in the previous part of this chapter. Yet in combination with the ‘horizontal’ elements of political power-sharing, federalism and decentralisation may become more effective for minority voice and protection (Fleiner et al. 2003).
5. *Consensus democracy rejects the hegemonic claims of a single group and avoids the fallacy of a monocultural nation-state*. Consensus democracy is viable only under conditions of recognition of equality of all societal cultures and their groups before the state. Thus, political power-sharing requires a certain acceptance of societal and cultural pluralism. This pluralism must be instilled into the basic concept of the state: the latter must guarantee equal rights to *all* its citizens and renounce on undue privileges for a specific culture and thus discriminate others. In contrast to the cultural or ‘ethnic nation’, this amounts to a political or ‘civic’ conception of the nation (*Verfassungspatriotismus*, for Habermas 1992), where citizenship is the only qualification for membership. Such a concept is basically indifferent to the religion, language or ethnicity of its different groups. Of course, every constitutional order, to a certain degree, is characterised by the heritage of a specific culture and its predominant values. The idea of separation of religion and the state, for instance, is realised in different ways and to different degrees in industrialised Western democracies (Madeley and Enyedi 2003). These differences may be greater still in developing societies where ligatures of religion are much stronger. Non-industrialised, traditional societies exposed to outside pressure of accelerating modernisation are sometimes even pushed towards relying on religion and other cultural traditions. However, values that symbolise a precious good for one cultural segment may be threatening for another. Such divides can be overcome only by the development of equal rights, mutual respect among all cultural groups and the development of common—or at least neutral—values. Such a collective identity or political culture requires a high degree of indifference or impartiality on the part of state authorities towards particular cultures.
6. *The development of a* *political culture* *of power-sharing takes time*. A new constitution can be written in a few weeks, political parties founded, elections held, and a parliament and government installed in a few years. Successful democratisation, however, takes much longer because the consolidation of institutions, the functioning of the political process, and the appropriate behaviour of actors all necessitate the development of a democratic political culture. In times of global pressure towards accelerated modernisation and quick conflict intervention by the international community, it should not be forgotten that changes in social values, the development of common views among different segments and cultural pluralism are processes of social integration that take time. Even more patience is needed when it comes to power-sharing as a means to overcome societal divides and accommodate deep social conflicts. The wounds of discrimination and civil war take generations to heal (Esman 1990, 14ff.). More than majoritarian settings, power-sharing institutions incite a ‘spirit of accommodation’ (Lijphart 1968, 104), respect, trust or even ‘deliberative potentials’ (Steenbergen 2009, 287). But these incentives cannot be accelerated, are even weak and vulnerable. While trust in consensus democracy takes a long time to develop, it may quickly be destroyed by the hegemonic use of power.
7. *Consensus democracy provides better chances, but still no guarantee for the peaceful resolution of conflict in multicultural societies*. Peaceful conflict resolution in deeply divided societies depends on many circumstances: on the economy and resources, neighbours and foreign interests, on culture and history—and on the political institutions. The latter are just one of many factors. The only proposition here is made with regard to the type of democracy: if the choice is between majoritarian and consensus institutions, the latter provide better chances for the resolution of multicultural conflict. In theory, there are two major arguments against consensus democracy. First, it is said that the political will to share power depends to a great extent on political elites, and that power-sharing can turn into an elitist model of democracy. Second, consensus democracy can be used by hegemonic groups as a veil to hide their real power in giving minorities the opportunity to participate but no substantial influence (McRae 1990). In this case, which can be observed for instance in the relations between the Jewish majority and the Arab minority in Israel, Ian Lustick (1980) speaks of a ‘control model’, with characteristics entirely different from the consensus model. Neither argument devalues the consensus model as such—but they illustrate its limits: the consensus model offers better chances or opportunities than majoritarian democracy, yet there is no guarantee that a successful political integration through mutual adjustment will actually occur.

## Footnotes

1 Separate aspects to consider are the existence of a quorum and/or the type of majority required. In fact, all four types may exist with or without a quorum and necessitate simple, qualified or compound majorities (cf. Altman 2019, 8).

2 See https://www.sos.ca.gov/elections/ballot-measures [10.12.2020].

3 Even if, due to the winner-take-all rule within States, twice in the last 20 years a president got elected with *less* popular votes than his direct rival (George W. Bush in 2000 and Donald J. Trump in 2016).

4 Alternatively called ‘regionalization’ or ‘devolution’.

5 See the many scholarly contributions analysing subnational policy diffusion and/or intercantonal cooperation, for example, Bochsler (2009), Fischer and Jager (2020), Füglister (2012), Füglister and Wasserfallen (2014), Gilardi and Füglister (2008), Sager and Rielle (2013), Schaltegger (2004), Schnabel and Mueller (2017), Stadter (2018), Strebel (2011), Strebel and Widmer (2012).

6 See, for instance, https://english.elpais.com/elpais/2020/01/07/inenglish/1578391109_970993.html. Accessed 1 April 2020.

7 See also Steiner (1974). For a recent comparative discussion of consociationalism, see the December 2019 special issue of the *Swiss Political Science Review:* ‘Half A Century of Consociationalism—Cases and Comparisons’ (Bogaards and Helms 2019).

## References

[CR1] Adeney K, Swenden W (2019). Power-Sharing in the World’s Largest Democracy: Informal Consociationalism in India (and Its Decline?). Swiss Political Science Review.

[CR2] Altman D (2019). *Citizenship and Contemporary: Direct Democracy*.

[CR3] Anderson G (2008). *Federalism: An Introduction*.

[CR4] Aubert J-F, Mahon P (2003). *Petit commentaire de la Constitution Fédérale de la Confédération Suisse du 18 Avril 1999*.

[CR5] Auer A (1989). *Le référendum et l’initiative populaire aux Etats-Unis*.

[CR6] Bächtiger A, Spörndli M, Steenbergen M, Steiner J (2005). The Deliberative Dimension of Legislatures. Acta Politica.

[CR7] Barber BR (1984). *Strong Democracy. Participatory Politics for a New Age*.

[CR9] Bochsler D (2009). Neighbours or Friends? When Swiss Cantonal Governments Co-operate with Each Other. Regional & Federal Studies.

[CR10] Bogaards, Matthijs, and Ludger Helms, eds. 2019. Half a Century of Consociationalism – Cases and Comparisons. *Swiss Political Science Review* 25 (4): 341–574.

[CR11] Brennan J (2016). *Against Democracy*.

[CR12] Brown D, Institute of Intergovernmental Relations (2005). Who’s Afraid of Asymmetrical Federalism? A Summary Discussion. *2005 Special Series on Asymmetric Federalism*.

[CR13] Bühlmann M, Kriesi H (2007). *Political Participation: Quantity Versus Quality*.

[CR14] Butler D, Ranney A (1978). *Referendums. A Comparative Study of Practice and Theory*.

[CR15] C2D – Centre for Research on Direct Democracy. 2019. http://c2d.ch. Accessed 1 November 2019.

[CR16] Carothers T (1999). *Aiding Democracy Abroad*.

[CR17] Cheneval F, el-Wakil A (2018). The Institutional Design of Referendums: Bottom-Up and Binding. Swiss Political Science Review.

[CR18] Christmann A, Danaci D (2012). Direct Democracy and Minority Rights: Direct and Indirect Effects on Religious Minorities in Switzerland. Politics and Religion.

[CR19] Coakley J (2017). *Non-Territorial Autonomy in Divided Societies*.

[CR20] Croly H (1914). *Progressive Democracy*.

[CR21] Cronin TE (1989). *Direct Democracy, The Politics of Initiative, Referendum, and Recall*.

[CR22] Dahl RA (1989). *Democracy and Its Critics*.

[CR23] Delley J-D (1999). *Démocratie directe et politique étrangère en Suisse*.

[CR24] Deschouwer K (2012). *The Politics of Belgium: Governing a Divided Society*.

[CR25] Deutsch K (1976). *Die Schweiz als paradigmatischer Fall politischer Integration*.

[CR26] Downs A (1957). *An Economic Theory of Democracy*.

[CR27] Dryzek JS (2002). *Deliberative Democracy and Beyond: Liberals, Critics, Contestations*.

[CR28] Duchacek I (1985). Consociational Cradle of Federalism. Publius: The Journal of Federalism.

[CR29] Ekman J, Amnå E (2012). Political Participation and Civic Engagement: Towards a New Typology. Human Affairs.

[CR30] Elazar DJ (1985). Federalism and Consociational Regimes. Publius: The Journal of Federalism.

[CR31] Esman MJ, Montville JV (1990). Political and Psychological Factors in Ethnic Conflict. *Conflict and Peacemaking in Multiethnic Societies*.

[CR32] Fischer, Manuel, and Nicolas W. Jager. 2020. How Policy-Specific Factors Influence Horizontal Cooperation among Subnational Governments: Evidence from the Swiss Water Sector. *Publius: The Journal of Federalism*. 10.1093/publius/pjaa002.

[CR33] Fleiner T, Kälin W, Linder W, Saunders C, Blindenbacher R, Koller A (2003). Federalism, Decentralisation and Conflict Management in Multicultural Societies. *Federalism in a Changing World – Learning from Each Other*.

[CR34] Füglister K (2012). *Policy Laboratories of the Federal State? The Role of Intergovernmental Cooperation in Health Policy Diffusion in Switzerland*.

[CR35] Füglister K, Wasserfallen F (2014). Swiss Federalism in a Changing Environment. Comparative European Politics.

[CR37] Gilardi F, Füglister K (2008). Empirical Modeling of Policy Diffusion in Federal States: The Dyadic Approach. Swiss Political Science Review.

[CR38] Gilardi F, Wasserfallen F (2016). How Socialization Attenuates Tax Competition. British Journal of Political Science.

[CR39] Giudici A, Stojanović N (2016). Die Zusammensetzung des Schweizerischen Bundesrates nach Partei, Region, Sprache und Religion, 1848–2015. Swiss Political Science Review.

[CR40] Gurr TR (2000). *Peoples Versus States: Minorities at Risk in the New Century*.

[CR41] Habermas J (1992). *Faktizität und Geltung*.

[CR42] Häfelin U, Haller W, Keller H, Turnheer D (2016). *Schweizerisches Bundesstaatsrecht*.

[CR43] Hehir A (2009). Independence, Intervention and Great Power Patronage: Kosovo, Georgia and the Contemporary Self-determination Penumbra. Amsterdam Law Forum.

[CR44] Hofstadter R (1955). *The Age of Reform*.

[CR45] Hooghe L, Marks G, Schakel AH, Niedzwiecki S, Chapman Osterkatz S, Shair-Rosenfield S (2016). *Measuring Regional Authority: A Postfunctionalist Theory of Governance*.

[CR46] Iff A (2009). *Peace Preserving Federalism – Making Sense of India and Nigeria*.

[CR47] Iff A, Töpperwien N, Federal Department of Foreign Affairs (2008). Power Sharing – The Swiss Experience. *Politorbis: Zeitschrift zur Aussenpolitik*.

[CR48] Jans MT, Coppieters B (2000). Personal Federalism: A Solution to Ethno-National Conflicts? What It Has Meant in Brussels and What It Could Mean in Abkhazia. *Federal Practice. Exploring Alternatives for Georgia and Abkhazia*.

[CR49] Kälin W (1986). Verfassungsgrundsätze der schweizerischen Aussenpolitik. Zeitschrift für Schweizerisches Recht.

[CR50] Kälin W, Bächler G (1997). Federalism and the Resolution of Minority Conflicts. *Federalism Against Ethnicity? Institutional, Legal and Democratic Instruments to Prevent Violent Minority Conflicts*.

[CR51] Kälin W, Swiss Agency for Development and Cooperation (1999). Decentralisation – Why and How?. *Decentralization and Development*.

[CR52] Kelemen, Daniel. 2002. Globalization, Federalism and Regulation. In *Dynamics of Regulatory Change: How Globalization Affects National Regulatory Policies*, ed. UCIAS, vol. 1, Article 8.

[CR53] Kriesi H (2005). *Direct Democratic Choice: The Swiss Experience*.

[CR54] Kriesi H, Vatter A (2009). Sind Abstimmungsergebnisse käuflich?. *Demokratie als Leidenschaft*.

[CR55] Ladner A, Keuffer N, Baldersheim H, Hlepas N, Swianiewicz P, Steyvers K, Navarro C (2019). *Patterns of Local Autonomy in Europe*.

[CR56] Lake D, Rothchild D (1998). *The International Spread of Ethnic Conflict*.

[CR57] Leftwich A, Leftwich A (1996). On the Primacy of Politics in Development. *Democracy and Development: Theory and Practice*.

[CR58] Lemarchand R, Bächler G (1997). Ethnic Conflict in Contemporary Africa. Four Models in Search of Solution. *Federalism Against Ethnicity? Institutional, Legal and Democratic Instruments to Prevent Violent Minority Conflicts*.

[CR59] Lijphart A (1968). *The Politics of Accommodation: Pluralism and Democracy in the Netherland*s.

[CR60] Lijphart A (1969). Consociational Democracy. World Politics.

[CR61] Lijphart A (1977). *Democracy in Plural Societies: A Comparative Exploration*.

[CR62] Lijphart A (1984). *Democracies, Patterns of Majoritarian and Consensus Government in Twenty-One Countries*.

[CR63] Lijphart A (1999). *Patterns of Democracy: Government Forms and Performance in Thirty-Six Countries*.

[CR64] Lijphart A (2008). *Thinking About Democracy, Power Sharing and Majority Rule in Theory and Practice*.

[CR65] Lijphart A (2012). *Patterns of Democracy: Government Forms and Performance in Thirty-Six Countries*.

[CR66] Linder W, Bächler G (1997). Federalism and Power-Sharing as a Means to Prevent Internal Conflict. *Federalism Against Ethnicity? Institutional, Legal and Democratic Instruments to Prevent Violent Minority Conflicts*.

[CR67] Linder W (2002). *Political Challenges of Decentralisation*.

[CR68] Linder W (2010). On the Merits of Decentralization in Young Democracies. Publius: The Journal of Federalism.

[CR69] Linder W, Bächtiger A (2005). What Drives Democratisation in Africa and Asia?. European Journal of Political Research.

[CR122] Linder, Wolf, and Sean Mueller. 2017. *Schweizerische Demokratie. Institutionen, Prozesse, Perspektiven*. 4th ed. Bern: Haupt.

[CR70] Linder W, Zürcher R, Bolliger C (2008). Gespaltene Schweiz – geeinte Schweiz. *Gesellschaftliche Spaltungen und Konkordanz bei den Volksabstimmungen seit 1874*.

[CR71] Litvack J, Junaid A, Bird R (1998). *Rethinking Decentralization in Developing Countries*.

[CR72] Loewenstein DH (1982). Campaign Spending and Ballot Propositions: Recent Experience, Public Choice Theory, and the First Amendment. UCLA Law Review.

[CR73] Lustick I (1980). *Arabs in the Jewish State: Israel’s Control of a National Minority*.

[CR74] Macpherson CB (1977). *The Life and Times of Liberal Democracy*.

[CR75] Madeley, John, and Zsolt Enyedi, eds. 2003. Church and State in Contemporary Europe. *West European Politics* 26 (1).

[CR76] McGlinchey M (2019). Does Moderation Pay in a Consociational Democracy? The Marginalisation of the SDLP in the North of Ireland. Swiss Political Science Review.

[CR77] McRae KD, Montville JV (1990). Theories of Power-Sharing and Conflict Management. *Conflict and Peacemaking in Multiethnic Societies*.

[CR78] Milanovic B (2016). *Global Inequality: A New Approach for the Age of Globalization*.

[CR80] Moore M (2001). Political Underdevelopment. What Causes “Bad Government”?. Public Management Review.

[CR81] Mueller S, Benz A, Sonnicksen J (2020). Federalism and Direct Democracy in Switzerland: Competing or Complementary?. *Federal Democracies at Work. Varieties of Complex Government*.

[CR82] Mukherjee B (2004). Why Power-Sharing Agreements Lead to Enduring Peaceful Resolution of Some Civil Wars, But Not Others?. International Studies Quarterly.

[CR83] Neidhart L (1970). *Plebiszit und pluralitäre Demokratie*.

[CR84] Nielsen CA (2009). The Kosovo Precedent and the Rhetorical Deployment of Former Yugoslav Analogies in the Cases of Abkhazia and South Ossetia. Southeast European and Black Sea Studies.

[CR85] Norris P (2008). *Driving Democracy: Do Power-Sharing Institutions Work?*.

[CR86] O’Leary B (2019). Consociation in the Present. Swiss Political Science Review.

[CR87] Oluvu D, Wunsch J (2004). *Local Governance in Africa – The Challenges of Decentralisation*.

[CR88] Paech N (2019). *Menschenrechte: Geschichte und Gegenwart, Anspruch und Realität*.

[CR89] Peters M (2019). Can Democracy Solve the Sustainability Crisis? Greenpolitics, Grassroots Participation and the Failure of the Sustainability Paradigm. Educational Philosophy and Theory.

[CR90] Przeworski AE, Alvarez M, Cheibub JA, Limongi F (2000). *Democracy and Development. Political Institutions and Well-Being in the World, 1950*–*1990*.

[CR91] Qvortrup M (2018). *Referendums Around the World*.

[CR92] Ra’anan U, Montville JV (1990). The Nation-State Fallacy. *Conflict and Peacemaking in Multiethnic Societies*.

[CR93] Remond, Alexandra. 2015. Power-Sharing in Africa: Does It Still Have a Role to Play? *E-International Relations*. https://www.e-ir.info/2015/07/01/power-sharing-in-africa-does-it-still-have-a-role-to-play/. Accessed 1 December 2019.

[CR94] Roeder P, Rothchild D (2005). *Sustainable Peace, Power and Democracy After Civil Wars*.

[CR95] Rosenberg SW (2007). *Deliberation, Participation and Democracy: Can the People Govern?*.

[CR96] Sager F, Rielle Y (2013). Sorting Through the Garbage Can: Under What Conditions Do Governments Adopt Policy Programs?. Policy Sciences.

[CR97] Schaltegger CA (2004). Finanzpolitik als Nachahmungswettbewerb: Empirische Ergebnisse zu Budgetinterdependenzen unter den Schweizer Kantonen. Swiss Political Science Review.

[CR98] Scharpf F (1970). *Demokratietheorie zwischen Utopie und Anpassung*.

[CR99] Schnabel J, Mueller S (2017). Vertical Influence or Horizontal Coordination? The Purpose of Intercantonal Conferences in Switzerland. Regional & Federal Studies.

[CR100] Schneider G, Hess C (1995). Die innenpolitische Manipulation der Aussenpolitik: Die Logik von Ratifikationsdebatten in der direkten Demokratie. Swiss Political Science Review.

[CR101] Schneider G, Weitsman P (1996). The Punishment Trap, Integration Referendums as Popularity Tests. Comparative Political Studies.

[CR102] Schumpeter J (1942). *Capitalism, Socialism, and Democracy*.

[CR103] Senghaas D, Senghaas D (1997). Frieden – Ein mehrfaches Komplexprogramm. *Frieden machen*.

[CR104] Sisk TD (1996). *Power Sharing and International Mediation in Ethnic Conflicts*.

[CR105] Stadter, Cornelia. 2018. *Policy Design, Innovation and Diffusion: Evidence from Cantonal Public Health Policies in Switzerland*. PhD dissertation, University of Zurich. https://www.zora.uzh.ch/id/eprint/117434/1/20183383.pdf. Accessed 1 April 2020.

[CR106] Steenbergen M, Vatter A (2009). Deliberative Politics in Switzerland. *Demokratie als Leidenschaft*.

[CR107] Steiner J (1974). *Amicable Agreement Versus Majority Rule: Conflict Resolution in Switzerland*.

[CR108] Strebel F (2011). Inter-Governmental Institutions as Promoters of Energy Policy Diffusion in a Federal Setting. Energy Policy.

[CR109] Strebel F, Widmer T (2012). Visibility and Facticity in Policy Diffusion: Going Beyond the Prevailing Binarity. Policy Sciences.

[CR110] Szczerbiak, Aleks, and Paul Taggard, eds. 2004. Choosing Union: The 2003 EU Accession Referendums. *West European Politics* 27 (4).

[CR111] Tarrow S, della Porta D (2005). *Transnational Protest and Global Activism*.

[CR112] *The Economist*. 15 May 2009.

[CR113] Thürer, Daniel, and Thomas Burri. 2009. Secession. Max Planck Encyclopedia of Public International Law. https://opil.ouplaw.com/view/10.1093/law:epil/9780199231690/law-9780199231690-e1100. Accessed 1 December 2019.

[CR114] Vatter A (2018). *Swiss Federalism: The Transformation of a Federal Model*.

[CR116] Walsh D (2018). *Territorial Self-Government as a Conflict Management Tool*.

[CR117] Wasserfallen F (2015). The Cooperative Capacity of Swiss Federalism. Swiss Political Science Review.

[CR118] Watts RL (2008). *Comparing Federal Systems*.

[CR119] White PG (2000). *Non-Territorial Federalism: A New Approach to Identity Politics*.

[CR120] Wimmer A (2018). *Nation Building: Why Some Countries Come Together While Others Fall Apart*.

[CR121] Zisk BH (1987). *Money, Media and the Grass Roots: State Ballot Issues and the Electoral Process*.

